# An updated meta-analysis of effects of curcumin on metabolic dysfunction-associated fatty liver disease based on available evidence from Iran and Thailand

**DOI:** 10.1038/s41598-023-33023-3

**Published:** 2023-04-10

**Authors:** Thitiya Lukkunaprasit, Amarit Tansawet, Suparee Boonmanunt, Abhasnee Sobhonslidsuk, Gareth J. McKay, John Attia, Ammarin Thakkinstian

**Affiliations:** 1 Department of Pharmacy Administration, College of Pharmacy, Rangsit University, Pathum Thani, Thailand; 2 Department of Surgery, Faculty of Medicine Vajira Hospital, Navamindradhiraj University, Bangkok, Thailand; 3 Department of Clinical Epidemiology and Biostatistics, Faculty of Medicine Ramathibodi Hospital, Mahidol University, Bangkok, Thailand; 4 Division of Gastroenterology and Hepatology, Department of Medicine, Faculty of Medicine Ramathibodi Hospital, Mahidol University, Bangkok, Thailand; 5 Centre for Public Health, School of Medicine, Dentistry, and Biomedical Sciences, Queen’s University Belfast, Belfast, UK; 6 School of Medicine and Public Health, University of Newcastle, Newcastle, Australia; 7 Hunter Medical Research Institute, New Lambton Heights, Australia

**Keywords:** Non-alcoholic fatty liver disease, Medical research

## Abstract

Metabolic dysfunction-associated fatty liver disease (MAFLD) is a common cause of chronic liver disease and can progress to nonalcoholic steatohepatitis and cirrhosis. This study aims to summarize the evidence for the effects of curcumin on MAFLD progression. Studies were identified from Medline and Scopus databases until April 2022. Systematic reviews and meta-analyses (SRMA) and randomized controlled trials (RCT) were selected based on pre-specified criteria. Three reviewers independently extracted data and assessed quality of included studies. Of the 427 identified records, 6 SRMAs and 16 RCTs were included in the analysis. Very high overlap was observed among SRMAs with corrected covered area of 21.9%. From an updated meta-analysis, curcumin demonstrated significant improvement in aspartate and alanine aminotransferase with pooled mean difference [95% confidence interval (CI)] of −3.90 (−5.97, −1.82) and −5.61 (−9.37, −1.85) units/L, respectively. Resolution and improvement of hepatic steatosis was higher in curcumin than control group with pooled relative risk (95% CI) of 3.53 (2.01, 6.22) and 3.41 (1.36, 8.56), respectively. Curcumin supplementation also led to lower fasting blood sugar, body mass index, and total cholesterol. Further trials should be conducted to assess the effect of curcumin on liver histology, especially regarding non-invasive hepatic fibrosis and steatosis.

## Introduction

Nonalcoholic fatty liver disease (NAFLD) is one of the most common causes of chronic liver disease^1^, and its incidence is increasing across the world along with greater metabolic dysfunction, including hypertension, atherosclerosis, type 2 diabetes (T2D), and obesity^2–4^. The spectrum of NAFLD consists of two subtypes: nonalcoholic fatty liver (NAFL) and nonalcoholic steatohepatitis (NASH)^4^. Recent studies highlighted that patients with both NAFL and NASH are at increased risk for disease progression^4^ with up to 25% of NAFLD cases progressing to advanced fibrosis or cirrhosis, and 7% to end-stage liver disease^4^ with the only curative option for the latter involving liver transplantation.

Given improved understanding of the pathogenesis of NAFLD, an international panel of experts has proposed new terminology around the term metabolic dysfunction-associated fatty liver disease (MAFLD)^5,6^, which is based on evidence of hepatic steatosis, along with one or more of the following conditions: obesity/overweight, T2D, or laboratory results representing metabolic dysregulation^5,7^. The pathophysiology of MAFLD and NASH is complex, involving multiple environmental exposures and genetic predisposition^8^. The accumulation of free fatty acid in the liver from the systemic circulation, de novo hepatic lipogenesis, and dietary lipids, all contribute to hepatic steatosis^8^. Stimulation of hepatic stress mechanisms, followed by oxidative stress, mitochondrial dysfunction, stellate cell activation, and intestinal dysbiosis, aggravate the pathogenesis of MAFLD and NASH^8^. The degree of liver fibrosis can be established by liver biopsy, which is the gold standard for diagnosis and severity assessment; however, this method is invasive and susceptible to sampling error. New imaging modalities (e.g., magnetic resonance elastography (MRE) and transient elastography (FibroScan®) focus on measuring liver stiffness, a surrogate marker for liver fibrosis.

Despite increasing prevalence of MAFLD globally, there has been no approved pharmacotherapy for this condition^9,10^. Curcumin, a polyphenol found in turmeric, has been reported to have antioxidant, anti-inflammatory, hepatoprotective and anti-atherosclerotic effects^11,12^ in metabolic syndrome, MAFLD, T2D, and polycystic ovarian syndrome, etc. Several systematic reviews and meta-analyses (SRMAs) have summarized the effects of curcumin in MAFLD patients^13–17^. However, the results relating to liver enzymes are still controversial, with some meta-analyses (MAs) demonstrating positive results^13,16^ while others report negative effects of curcumin on aspartate aminotransferase (AST) and/or alanine aminotransferase (ALT)^14,15,17^. Furthermore, no SRMAs have investigated liver fat content or liver stiffness. This review was conducted to summarize and update the effects of curcumin on the progression of MAFLD.

## Methods

This review was conducted following the Preferred Reporting Items for Systematic Review and Meta-Analysis (PRISMA) guidelines^18^ and was registered at PROSPERO (CRD42022323057).

### Search strategies

Studies were identified from Medline via PubMed and Scopus databases up to April 2022. In addition, reference lists of identified studies were searched. Search terms were constructed based on only population (P) and intervention (I) domains. Population was defined as adult patients with MAFLD (i.e., NAFLD or NASH) with or without comorbidity (e.g., T2D, obesity, etc.). Intervention was any form of curcumin supplement including curcumin extracts and turmeric powder with a placebo or standard treatment comparator. These search terms were combined within and between domains using conjunctions ‘OR’ and ‘AND’, respectively. The search terms are listed in Supplementary Appendix A. The study designs included SRMA for the umbrella review and randomized controlled trials (RCT) for the updated MA.

### Study selection

SRMAs published in English or other languages translatable using Google Translate were selected if they met the following inclusion criteria: SRMAs of RCTs of adults with MAFLD or NASH, comparisons between any form of supplemented curcumin (e.g., curcumin or turmeric powder, *Curcuma domestica*, *Curcuma longa*, curcuminoid) and placebo or only standard treatment, and pooled any outcome of interest indicated below. For updated pooling by MA, individual RCTs were selected if they included adults with MAFLD, compared curcumin supplement with placebo or standard treatment, and reported any of the following outcomes: liver function tests, resolution/improvement of hepatic steatosis by ultrasonography (US), or liver stiffness, and steatosis by elastography. Studies were excluded if they duplicated publications of the same studies, their full-texts could not be retrieved, or published in non-English languages that could not be translated by Google translator. Three reviewers (ATa, SB, TL) independently selected studies. Disagreements were discussed and resolved by consensus.

### Outcomes of interest

The outcomes of interest were liver enzymes [i.e., ALT, AST, alkaline phosphatase (ALP), and gamma-glutamyl transpeptidase (GGT)], the resolution of hepatic steatosis (i.e., MAFLD grade 1–3 resolved to grade 0) or the improvement of hepatic steatosis (i.e., lowering of MAFLD grading after treatment) assessed with ultrasound, and liver stiffness and steatosis evaluated with transient elastography (known as FibroScan^®^). In addition, glycemic indices (i.e., HbA1c and fasting blood sugar: FBS), body mass index (BMI), lipid profile [i.e., total cholesterol (TC), triglycerides (TG), low-density lipoprotein cholesterol (LDL-C), and high-density lipoprotein cholesterol (HDL-C), blood pressure (i.e., systolic blood pressure (SBP) and diastolic blood pressure (DBP))], and other fibrosis indices [i.e., platelet count, Fibrosis-4 (FIB4), NAFLD Fibrosis Score (NFS), the AST-to-platelet ratio index (APRI), and the BMI, AST/ALT ratio, and diabetes (BARD) score] were also assessed.

### Data extraction

Three reviewers (ATa, SB, TL) independently extracted data from the published studies. Any difference in data extracted was discussed and resolved by consensus. The following data were extracted: publication year, end search, number of included studies, participant characteristics (gender and age), type of patients (i.e., MAFLD/NASH), interventions, follow-up time, outcomes and methods of synthesis.

In addition, outcomes from the SRMAs were extracted, including number of studies, number of participants, pooled effect size (ES) with 95% confidence interval (CI) for each outcome, heterogeneity diagnostics (I^2^), and conflict of interest. Lastly, information regarding individual RCTs in each SRMA was extracted (first author and year) to construct a study-citation matrix across SRMAs.

### Quality assessment

The Risk of Bias in Systematic Reviews (ROBIS) checklist^19^ was used for umbrella reviews considering four domains, i.e., study eligibility criteria, methods for identification and selection of studies, data collection and study appraisal, data synthesis and findings. The results were graded as low or high risk of bias, if there was sufficient information to assess; otherwise, the results were graded as unclear.

For individual RCTs included in the updated MA, the Cochrane risk-of bias-tool for randomized trials (RoB 2)^20^ was applied to evaluate bias arising from the randomization process, deviations from intended interventions, missing outcome data, measurement, and findings reported. Overall judgment was low or high risk of bias or some concerns.

### Statistical analysis

The SRMAs findings were described for each outcome separately. The original effect sizes including mean difference (MD) and standardized mean difference (SMD) along with 95% CIs were described in a summary table and forest plots where appropriate. The degree of study overlap was estimated from a study-citation matrix by covered area (CA) and corrected covered area (CCA), and was categorized as slight (0–5%), moderate (6–10%), high (11–15%) or very high (> 15%).

For the integrated and updated MA, the efficacy of supplemented curcumin and control were directly compared and pooled for each outcome if at least two studies provided a comparison. MD and risk ratio (RR) were estimated for continuous outcomes (e.g., liver function test and fat content) and dichotomous outcome (US resolution), respectively. These effect measures were pooled across studies using a random-effect model if heterogeneity was present, otherwise a fixed-effect model was applied. Heterogeneity was assessed using Cochrane’s Q test and I^2^ statistics, and considered present if p-value < 0.10 or I^2^ > 25%. Source of heterogeneity was explored by fitting covariables [e.g., dose and form of curcumin (whole compounds, curcumin extracts, and bioavailability-enhanced forms), follow-up time, etc.] individually within a meta-regression model; with subgroup analyses performed accordingly. Publication bias was assessed using a funnel plot and Egger’s test. If asymmetry was detected, a contour enhanced funnel plot was constructed to identify the cause of asymmetry. All analyses were performed using STATA software, version 17. A p-value < 0.05 was considered statistically significant.

## Results

### Umbrella review

#### Identification and selection of SRMAs and individual studies

**Figure 1:**
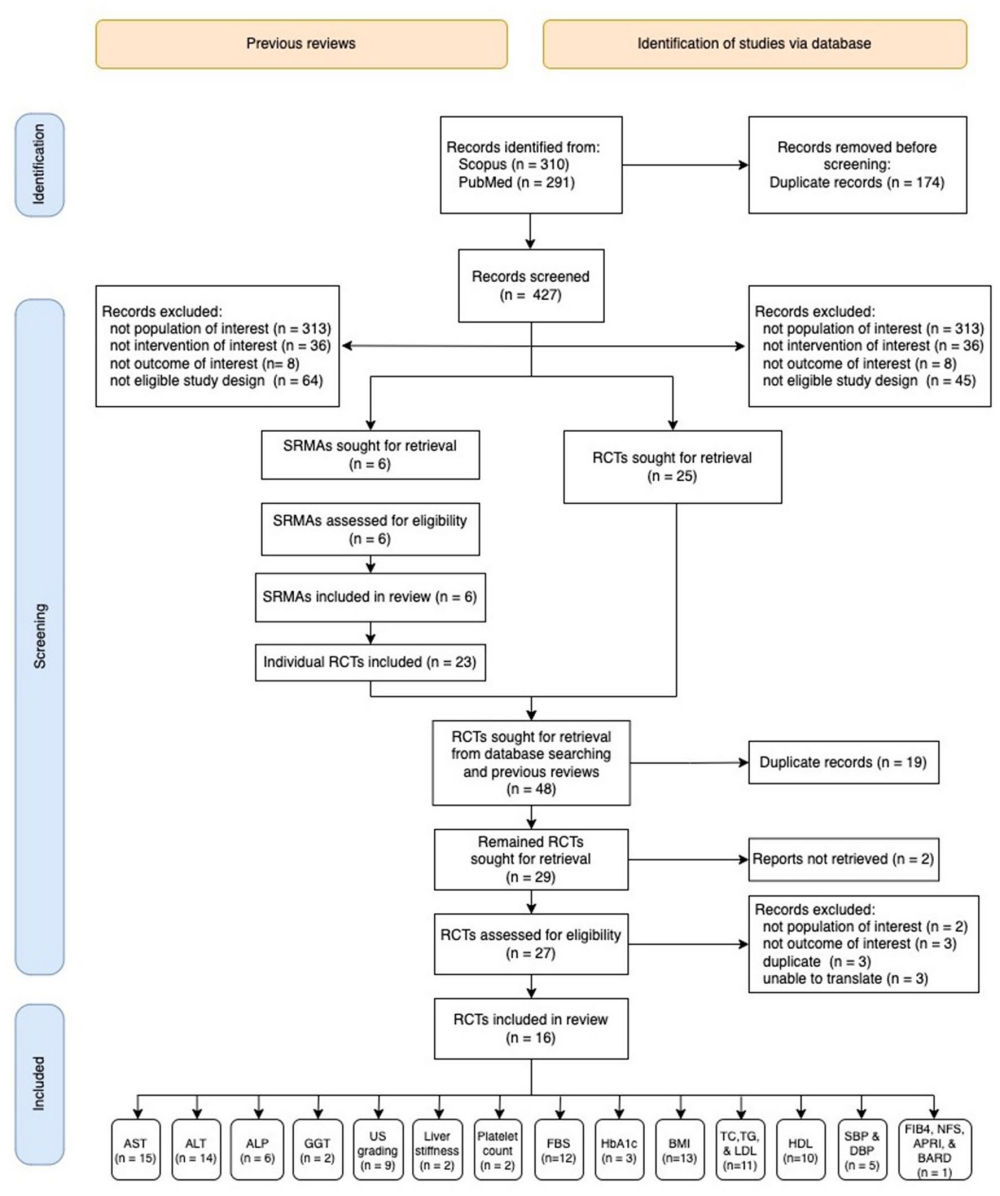
The PRISMA flow diagram visually summarizing the screening process.

Of the 427 studies identified from PubMed and Scopus, six previous SRMAs met the eligibility criteria and were included in the umbrella review, see Fig. 1. Of these, 23 RCTs were included in the SRMAs; these were then combined with 25 individual RCTs identified from our additional searches. After removing duplicates and two RCTs that could not be retrieved, 27 RCTs were assessed for eligibility, and 16 were included in the updated MA.

#### Description of SRMAs

Of the six SRMAs, five studies were direct MAs^13–16,21^ and one was a network meta-analysis (NMA)^17^. These studies were published between 2019 and 2022 and included 4 to 15 RCTs with a total sample size of 228 to 934 patients (see Table 1). Regarding the outcomes of interest, all SRMAs pooled effect sizes for AST and ALT by pooling MD^13–16^ and SMD^17,21^. Only one SRMA also pooled GGT, ALP, and improvement in NASH or fibrosis^17^. All studies used both curcumin extracts and turmeric powder^13–15,17,21^, with the exception of a single study^16^ that used only the curcumin extract form. The follow-up time ranged from 4 to 24 weeks.

**Table 1:** Baseline characteristics of systematic reviews and meta-analyses included.

No	First author, year	Country	End search	N (studies)	n (patients)	Female (%)	Mean age (years)	Patients	Intervention	Outcome	Measure	Follow up time	Conflict of interest
Pairwise meta-analysis													
1	Goodarzi, 2019	Iran	20 Nov 2018	6	315	50.5	40–65	NAFLD	Turmeric/curcumin	**AST, ALT**	MD	8, 12, 24 weeks	No COI
2	Mansour-Ghanaei, 2019	Iran	Dec 2017	4	228	NR	Curcumin: 42.1–66.7 Placebo: 40.4–64.4	NAFLD/NASH	Turmeric/curcumin	**AST, ALT**	MD	8, 12 weeks	No COI
3	Wei, 2019	China	Mar 2018	4	229	48.0	Curcumin: 42.09–52.70 Placebo: 40.38–56.67	NAFLD/NASH	Turmeric/curcumin	**AST, ALT**, TC, LDL, HDL, TG, FBS, HbA1c, insulin, HOMA-IR, weight	MD	8, 12, 24 weeks	No COI
4	Jalali,2020	Iran	1 Sep 2019	9	588	46.4	Curcumin: 41.8–46.64 Placebo: 37.75–48.95	NAFLD	Curcumin only	**AST, ALT**, LDL, HDL, TC, TG, FBS, HbA1c, insulin, HOMA-IR, weight, WC, BMI	MD	8, 12 weeks	No COI
5	Khalili, 2022	Iran	April 2021	14	714	NR	NR	NAFLD	Turmeric/curcumin	**AST, ALT**, TC, LDL, HDL, TG, FBS, BMI	SMD	4, 8, 12 weeks	No COI
Network meta-analysis													
6	Zhou, 2021	China	14 Aug 2020	35 (15 curcumin trials)	5246 (934 in curcumin trials)	NR	Curcumin: 40.95–66.72 Placebo: 40.06–64.36	NAFLD/NASH	**Turmeric/curcumin**, obeticholic acid, elafibranor, cenicriviroc, selonsertib, silymarin, resveratrol	Liver pathology improvement, **AST, ALT**, GGT, ALP, TC, TG, LDL, HDL	Exponential SMD	8 weeks to 2 years (8–12 weeks for curcumin trials)	No COI

*ALT* alanine aminotransferase, *ALP* alkaline phosphatase, *AST* aspartate aminotransferase, *BMI* body mass index, *COI* conflict of interest, *FBS* fasting blood sugar, *GGT* gamma glutamyl transpeptidase, *HbA1c* hemoglobin A1c, *HDL* high-density lipoprotein, *HOMA-IR* homeostatic model assessment of insulin resistance, *LDL* low-density lipoprotein, *MD* mean difference, *NAFLD* nonalcoholic fatty liver disease, *NASH* nonalcoholic steatohepatitis, *NR* not reported, *SMD* standardized mean difference, *TC* total cholesterol, *TG* triglyceride.

Significant values are in bold.

#### Methodological quality of included systematic reviews

Supplementary Appendix B summarizes the methodological quality of all six SRMAs included. Two SRMAs (33.33%) were considered at low risk of bias^13,15^, in contrast to four SRMAs (66.67%) that were considered high risk due to the identification and selection of studies^16^, data collection and study appraisal^17,21^, and synthesis and findings^14,16,17,21^.

#### Degree of overlap in SRMAs

A study-citation matrix was constructed to calculate the degree of overlap between the individual RCTs included in the pooling of AST and ALT across the SRMAs. The CCA score was 21.9% representing a very high degree of overlap. Fourteen of the 21 RCTs were included in multiple SRMAs, suggesting that each SRMA failed to add incremental information, see Supplementary Appendix C.

#### Liver enzymes

All six SRMAs reported changes of AST and ALT: four SRMAs^13–16^ pooled MD, whereas two SRMAs^17,21^ pooled SMD. For AST, the pooled MDs reported in four SRMAs ranged from −0.78 to −7.43 units/L with an I^2^ of 0 to 92%. However, only three^13–15^ of these four MDs, ranging from −4.68 to −7.43 were significant, suggesting curcumin could enable a small reduction in AST (about 5 to 7 units/L) compared to placebo, see Supplementary Appendix D. Two remaining SRMAs^17,21^ showed pooled SMDs ranging from −0.25 and −0.35, but these were not significant.

The pooled MDs of ALT ranged from 0.46 to 7.47 units/L with heterogeneity ranging from 0 to 82.3%. Of these, two MDs^13,16^ were statistically significant suggesting curcumin could reduce ALT by 0.46 to 7.47 units/L, see Supplementary Appendix E. Two other SRMAs^17,21^ showed no significant effect.

### Updated meta-analysis

Sixteen RCTs^22–37^ were included in the updated MA with the outcomes including AST (N = 15), ALT (N = 14), ALP (N = 6), GGT (N = 2), US grading (N = 9), liver stiffness (N = 2), platelet count (N = 2), FBS (N = 12), HbA1c (N = 3), BMI (N = 13), TC (N = 11), TG (N = 11), LDL-C (N = 11), HDL-C (N = 10), SBP (N = 5 ), DBP (N = 5), FIB4 (N = 1), NFS (N = 1), APRI (N = 1), and BARD (N = 1), see Fig. 1.

#### Baseline characteristics

Baseline characteristics for the studies included are shown in Table 2. All compared curcumin supplement to placebo. Most studies were conducted in Iran with the exception of a single study conducted in Thailand. Mean age ranged from 41.0 to 52.7 years and 37.8 to 56.7 years in curcumin and placebo groups, respectively. Gender was distributed similarly between the interventions in 4 of the 14 studies that provided information. Mean BMI ranged from 27.6 to 32.3 kg/m^2^ and 27.3 to 32.9 kg/m^2^ in both groups. Curcumin forms varied including bioavailability-enhanced forms of curcumin (80–1000 mg/day) in 11 RCTs, curcumin extracts (1500 mg/day) in two RCTs, and whole compounds, i.e., turmeric powder, (2000–3000 mg/day) in three RCTs. The bioavailability-enhanced forms included phytosomal curcumin (N = 5), curcumin combined with piperine (N = 3), nano-micellar curcumin (N = 2), and curcumin amorphous dispersion (N = 1). The study duration ranged from 8 to 24 weeks.

**Table 2:** Baseline characteristics for the randomized-controlled trials included within the updated meta-analysis.

Study	Country	Enrollment period	Patients’ characteristics (curcumin vs placebo)			Curcumin		Follow-up, weeks
			Age, years (mean ± SD)	% Female	BMI, kg/m^2^ (mean ± SD)	Form	Dose, mg/day	
Chirapongsathorn 2012	Thailand	Aug 2010–Aug 2011	52.7 ± 7.6 56.7 ± 14.6	30 55.6	29.7 ± 4.1 28.9 ± 3.3	Whole compound	NA	24
Rahmani 2016	Iran	NA	46.4 ± 11.6 49.0 ± 9.8	52.5 52.5	30.8 ± 4.5 31.4 ± 5.7	Amorphous dispersion	500	8
Navekar 2017	Iran	NA	42.1 ± 7.2 40.4 ± 9.3	47.6 61.9	31.8 ± 4.6 32.9 ± 4.8	Whole compound	3000	12
Panahi 2017	Iran	NA	45.0 ± 12.6 47.2 ± 10.3	45.5 37.2	29.0 ± 3.4 29.1 ± 3.5	Phytosomal curcumin	1000	8
Saadati 2018	Iran	Mar 2017–Aug 2017	46.6 ± 11.7 45.3 ± 11.5	56 42.9	NA	Curcumin extract	1500	12
Chashmniam 2019	Iran	Jan 2017–Aug 2017	46.6 ± 2.3 37.8 ± 3.2	48 30	30.0 ± 0.7 28.1 ± 0.9	Phytosomal curcumin	250	8
Jazayeri-Tehrani 2019	Iran	NA	41.8 ± 5.6 42.5 ± 6.2	45.2 45.2	30.7 ± 2.1 30.8 ± 2.4	Nanocurcumin	80	12
Mirhafez 2019a	Iran	NA	44.8 ± 11.1 40.7 ± 11.8	63.6 36.4	30.1 ± 5.8 27.7 ± 6.0	Phytosomal curcumin	250	8
Panahi 2019	Iran	NA	46.6 ± 2.2 47.5 ± 2.5	42.9 45.7	NA	Curcumin + Piperine	500	12
Saadati 2019a	Iran	NA	46.2 ± 11.5 45.1 ± 10.9	60.9 39.1	32.3 ± 4.6 32.4 ± 5.0	Curcumin extract	1500	12
Hariri 2020	Iran	Jan 2017—May 2018	41.0 ± 12.2 40.1 ± 13.7	47 53	30.6 ± 5.9 28.9 ± 3.6	Phytosomal curcumin	250	8
Moradi-Kelardeh 2020	Iran	NA	NA	NA	27.6 ± 1.3 27.3 ± 1.3	Nanocurcumin	80	12
Saberi-Karimian 2020	Iran	NA	NA	NA	30.0 ± 5.5 30.2 ± 4.1	Curcumin + Piperine	500	8
Jarahzadeh 2021	Iran	NA	44.1 ± 8.4 38.6 ± 10.4	40.6 40.6	29.5 ± 5.0 30.2 ± 5.1	Whole compound	2000	8
Mirhafez 2021a	Iran	Jan 2017–Aug 2017	45.0 ± 11.1 43.1 ± 11.6	45 40	30.8 ± 5.1 29.2 ± 4.2	Phytosomal curcumin	250	8
Mirhafez 2021c	Iran	Jan 2017–Aug 2017	45.6 ± 11.0 43.1 ± 11.6	46.2 40	30.9 ± 4.3 29.2 ± 4.2	Curcumin + Piperine	500	8

*NA* not available, *SD* standard deviation.

#### Risk of bias of RCTs included

The risk of bias was considered low for the outcomes measured and the selection of results reported, see Supplementary Appendix F and Supplementary Appendix G. However, half of the studies generated some concerns (43.8%) or high risk of bias (6.3%) given reported deviations from intended interventions, mostly due to the use of per-protocol analyses in their respective studies. Furthermore, 12.5% of the studies were represented by a high risk of bias in the randomization process and missing data outcomes. The overall risk of bias was rated as some concerns in 43.8% and high in 31.3% of studies.

#### Liver enzymes

### AST

**Figure 2:**
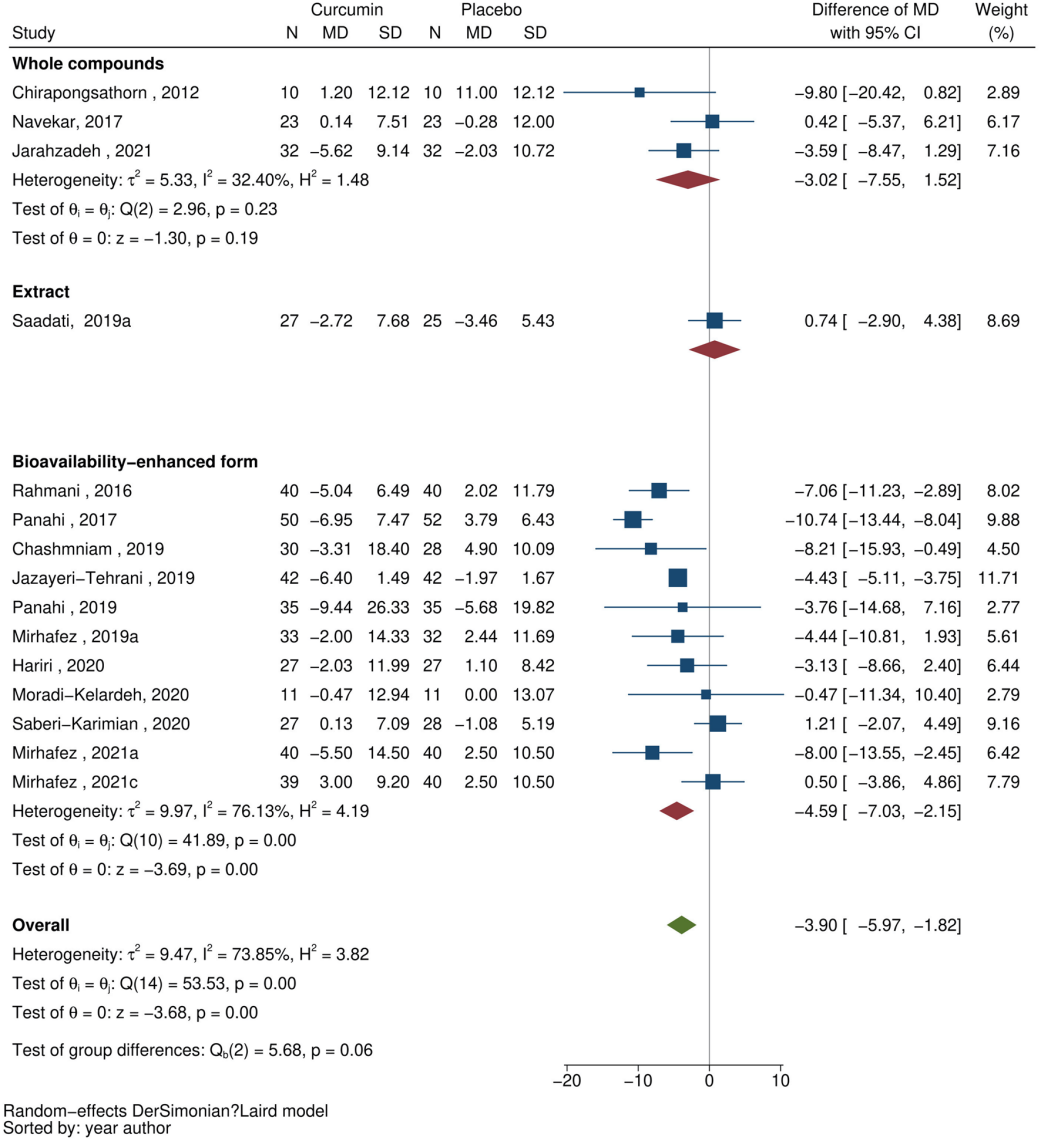
Forest plots demonstrating the effect of curcumin on aspartate aminotransferase: all curcumin forms.

Fifteen studies^22–25,27–37^ assessed the effect of curcumin supplement on AST, see Fig. 2. The overall pooling of data indicated that curcumin supplementation was significantly associated with reduced AST with MD (95% CI) of −3.90 (−5.97, −1.82) units/L, although high heterogeneity was observed (I^2^ = 73.9%). Fitting follow-up time in the meta-regression did not improve I^2^. Subgroup analysis was performed by curcumin forms, i.e., whole compounds (N = 3)^22,24,35^, curcumin extracts (N = 1)^31^, and bioavailability-enhanced forms (N = 11)^23,25,27–30,32–34,36,37^. The AST-lowering effect was stronger across all bioavailability-enhanced forms [MD (95% CI) of −4.59 (−7.03, −2.15) units/L], but not significant for whole compounds [MD (95% CI) of −3.02 (−7.55, 1.52) units/L]. However, heterogeneity was still high at 76.1% in the bioavailability-enhanced forms but lower for whole compounds (I^2^ = 32.4%).

The bioavailability-enhanced forms were further classified into phytosomal curcumin (N = 5)^25,27,29,32,36^, nano-micellar curcumin (N = 2)^28,33^, curcumin combined with piperine (N = 3)^30,34,37^, and curcumin amorphous dispersion (N = 1)^23^; sensitivity analyses by pooling the effects of these forms lowered heterogeneity to 0% to 49.1%, see Supplementary Appendix H. Phytosomal curcumin and nano-micellar curcumin were significantly associated with lowering AST level with MD (95% CI) of −7.42 (−10.68, −4.15) and −4.41 (−5.09, −3.74) units/L, respectively. However, the effect of curcumin-piperine was not significant on AST [MD (95% CI) of 0.70 (−1.85, 3.24) units/L].

### ALT

**Figure 3:**
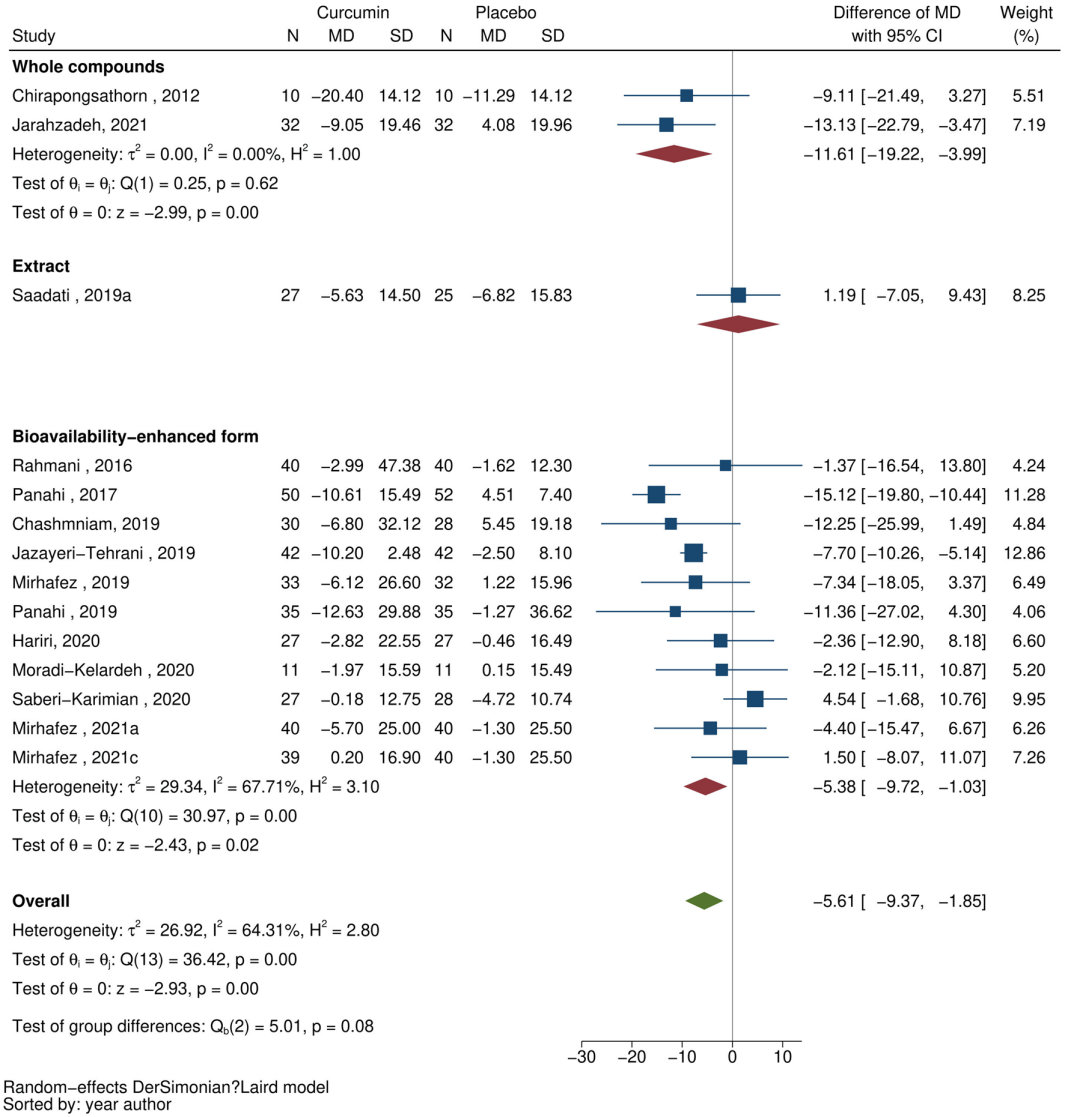
Forest plots demonstrating the effect of curcumin on alanine aminotransferase: all curcumin forms.

Fourteen studies^22,23,25,27–37^ evaluated the effect of curcumin supplementation on ALT, see Fig. 3. The overall pooling indicated that curcumin supplementation was significantly associated with reduced ALT [MD (95% CI) of −5.61 (−9.37, −1.85) units/L] with moderate heterogeneity observed (I^2^ = 64.3%). Fitting follow-up time in the meta-regression did not improve I^2^. In subgroup analyses, a similar effect and moderate levels of heterogeneity were still observed among the majority of studies that used bioavailability-enhanced forms [MD (95% CI) of −5.38 (−9.72, −1.03) units/L, I^2^ = 67.7%]. In contrast, studies using whole compounds doubled the effect on lower ALT [MD (95% CI) of −11.61 (−19.22, −3.99) units/L] with no heterogeneity observed (I^2^ = 0%).

Similarly, pooling the effects on ALT by bioavailability-enhanced forms in a sensitivity analysis reduced heterogeneity (I^2^ = 0% to 45.6%), and phytosomal curcumin and nano-micellar curcumin were significantly associated with lower ALT [MD (95% CI) of −9.29 (−15.02, −3.55) and −7.49 (−10.00, −4.98) units/L, respectively]. In contrast, the overall effect of curcumin-piperine was not significant [MD (95% CI) of 0.83 (−6.46, 8.12) units/L], see Supplementary Appendix I.

### ALP and GGT

Six studies^25,27,30,33,36,37^ reported the effect of curcumin supplement on ALP, see Supplementary Appendix J. There was no significant association between curcumin supplement and ALP [MD (95% CI) of −8.88 (−24.68, 6.93) units/L]. All studies included used different types of bioavailability-enhanced curcumin forms with a high degree of heterogeneity (I^2^ = 92.7%). For GGT, two studies^31,35^ were included but no significant effect was observed [MD (95% CI) of −3.87 (−10.66, 2.93) units/L] and levels of heterogeneity were low (I^2^ = 11.6%), see Supplementary Appendix K.

#### Hepatic steatosis by ultrasonography

**Figure 4:**
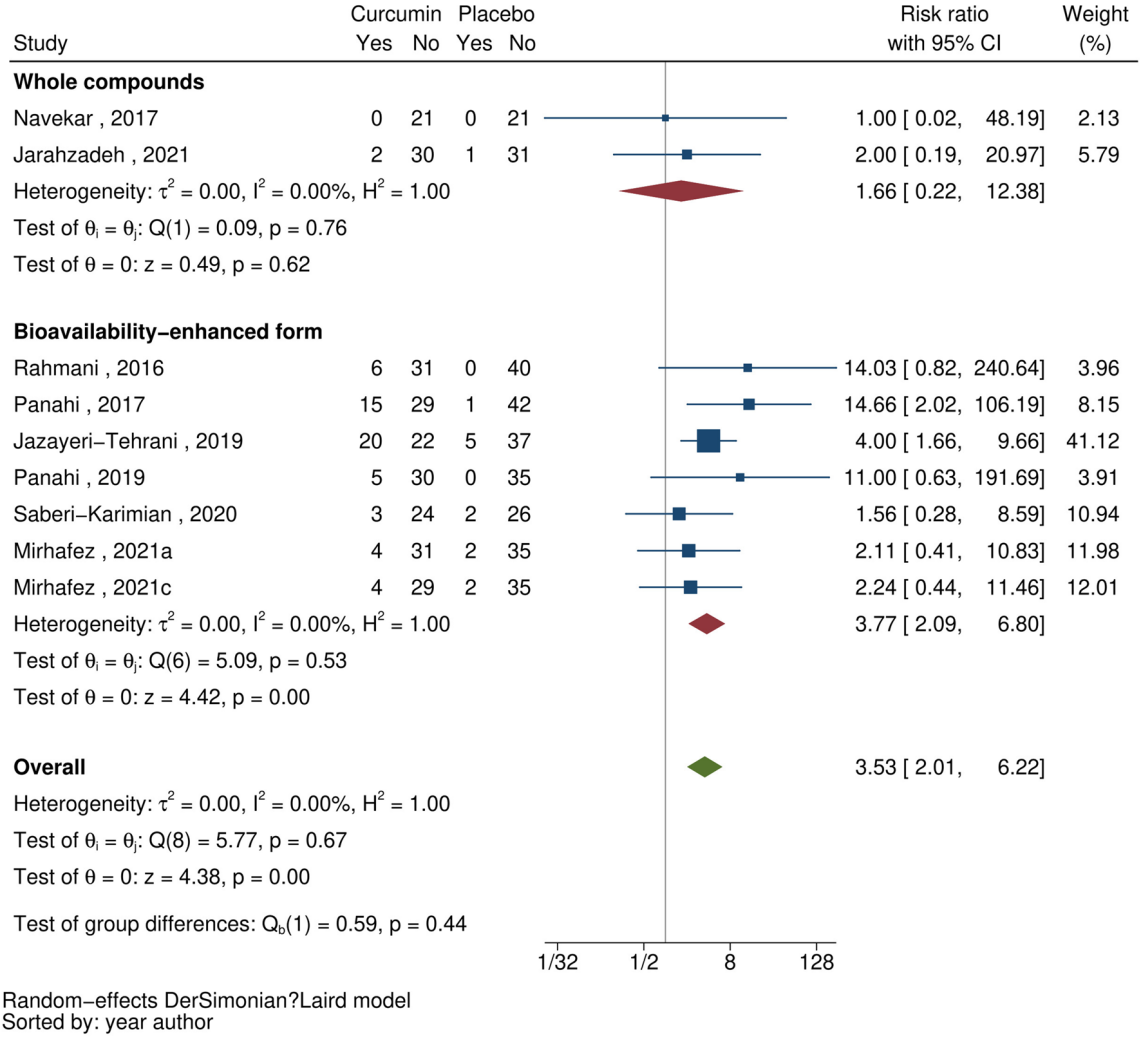
Forest plots demonstrating the effect of curcumin on the resolution of hepatic steatosis by ultrasonography.

Nine studies^23–25,28,30,34–37^ reported a change in MAFLD grade assessed by liver US after curcumin supplementation. A significant resolution of hepatic steatosis was observed following curcumin supplementation with an overall RR (95% CI) of 3.53 (2.01, 6.22) with no heterogeneity detected across studies (I^2^ = 0%), see Fig. 4. A slightly stronger effect was observed across the seven studies^23,25,28,30,34,36,37^ that used bioavailability-enhanced curcumin forms [RR (95% CI) of 3.77 (2.09, 6.80), I^2^ = 0%].

Changes in hepatic steatosis was also investigated across three studies^23,25,30^. Bioavailability-enhanced curcumin supplement was also significantly associated with improved hepatic steatosis with RR (95% CI) of 3.41 (1.36, 8.56), although high levels of heterogeneity were observed (I^2^ = 78.7%), see Supplementary Appendix L.

#### Liver stiffness

Liver stiffness was assessed with transient elastography and reported as a fibrosis score (kPa) in two studies^26,31^. The overall effect showed no significant association between curcumin supplementation and liver stiffness, with MD (95% CI) of −0.31 (−0.75, 0.13) kPa, I^2^ = 0%, see Supplementary Appendix M. Unfortunately, liver steatosis, represented by the controlled attenuation parameter of transient elastography, was reported in only one study^31^ and was not significant.

Platelet levels were assessed in two studies^30,33^. The pooled mean difference in platelet count between curcumin supplementation and placebo is not statistically significant with MD of 8.27 (−19.42, 35.95) × 10^9^/L, see Supplementary Appendix N. For other fibrosis indices, i.e., FIB4, NFS, APRI, and BARD, they were assessed in only one study^26^ thus these outcomes could not be pooled.

#### Glycemic indices

Twelve studies^22–24,27–31,34–37^ assessed reduction of FBS indicating curcumin supplementation was associated with a significant reduction in FBS, with MD (95% CI) of −2.05 (−3.08, −1.01) mg/dL, I^2^ = 0%, see Supplementary Appendix O. A significant effect persisted in bioavailability-enhanced curcumin with MD (95% CI) of −1.94 (−3.01, −0.87) mg/dL, I^2^ = 0%. However, curcumin was not significantly associated with HbA1c based on pooling three included studies^23,28,30^ with MD (95% CI) of −0.24 (−0.66, 0.18) %, I^2^ = 85.8%, see Supplementary Appendix P.

#### Body mass index (BMI)

Thirteen studies^23–29,31–34,36,37^ assessed reduction of BMI indicating curcumin supplementation was effective in lowering BMI with MD (95% CI) of −0.34 (−0.62, −0.05) kg/m^2^; I^2^ = 62.7%, see Supplementary Appendix Q. The effect is slightly stronger for curcumin in bioavailability-enhanced forms but with mildly higher heterogeneity across studies [MD (95% CI) of −0.44 (−0.78, −0.10) kg/m^2^; I^2^ = 67.4%].

#### Lipid profile

Eleven studies^22,23,27–31,34–37^ assessed changes in TC, TG, and LDL-C, while ten studies assessed the effect on HDL-C^23,27–31,34–37^. A significant reduction in TC was observed following curcumin supplementation with an overall MD (95% CI) of −8.18 (−14.07, −2.29) mg/dL with moderate heterogeneity across studies (I^2^ = 42.5%). The effect is slightly stronger when bioavailability-enhanced forms were used with MD (95% CI) of −10.04 (−17.55, −2.53) mg/dL and I^2^ of 50.0%, see Supplementary Appendix R. However, the pooled effects of curcumin supplementation on LDL, HDL, and TG were not statistically significant regardless of its forms, see Supplementary Appendix S–U.

#### Blood pressure

Five studies^25,28,34,36,37^ assessed changes in blood pressure indicating no significant effect of curcumin supplementation on both SBP and DBP with pooled MD (95% CI) of −0.29 (−0.91, 0.34) and −0.02 (−0.55, 0.52) mmHg, respectively, see Supplementary Appendix V–W.

#### Sensitivity analysis

Sensitivity analysis excluding studies with a high risk of bias was performed for AST, ALT, and the resolution of hepatic steatosis (by US). The AST- and ALT-lowering effects of curcumin were still significant with MD (95% CI) of −3.72 (−6.25, −1.19) and −5.62 (−10.57, −0.66) units/L, respectively, while the association between curcumin and the resolution of hepatic steatosis was slightly stronger with RR (95% CI) of 3.90 (2.01, 7.60), see Supplementary Appendices X–Z.

#### Publication bias

Publication bias was assessed by funnel plots and Egger’s test for all outcomes except GGT and liver stiffness due to the small number of pooled studies available. For AST, ALT, ALP, the resolution of steatosis, BMI, TC, and TG, the funnel plots were symmetrical and the Egger tests were not significant, suggesting no publication bias, see Supplementary Appendices AA–GG. For the improvement of hepatic steatosis, FBS, HbA1c, LDL-C, HDL-C, SBP, and DBP, the funnel plots were asymmetrical, however, the contour-enhanced funnel plots showed that the asymmetrical funnel plots were more likely caused by other reasons than publication bias, see Supplementary Appendices HH–TT. Heterogeneity might cause asymmetrical funnel plots of the improvement of hepatic steatosis, HbA1c, LDL-C, HDL-C, SBP, and DBP.

## Discussion

This study identified six meta-analyses of curcumin supplements for MAFLD/NASH patients. Although the degree of overlap was very high (CCA 21.9%), we still identified two RCTs^36,37^ that had not been included in previous meta-analyses because they were published after the end search of previous MAs. In addition, we also excluded two RCTs^38,39^, whose patients were not MAFLD but were mistakenly included in previous MAs. This allows prior findings to be updated. Significant but clinically small reductions in AST and ALT levels with curcumin were observed in our updated meta-analysis, consistent with previous reports. Reductions of GGT and ALP levels were also observed from curcumin supplementation but these were not significant. AST and ALT improvement may arise as a consequence of the antioxidative effects of curcumin which theoretically ameliorates liver cell injury^40^, in contrast to GGT and ALP, markers of cholestasis, which are less likely to benefit from antioxidant effects.

Anti-inflammatory effects of curcumin and related substances have been extensively studied in both in vitro and in vivo models. These studies suggested that curcumin may act on various pathways known to be associated with liver diseases such as TGF-β1/Smad, JNK1/2-ROS, NF-κB and other anti-inflammatory and antioxidant signaling pathways^41^. Curcumin might also induce activities of antioxidative stress enzymes (e.g., glutathione-linked detoxifying enzymes and heme oxygenase-1)^42,43^. Histological findings from studies using rodents showed that curcumin and related substances ameliorated liver fibrosis and reduced number of necrotic cells in a dose-dependent fashion^44–46^. Furthermore, curcumin might inhibit hepatic stellate cell proliferation, collagen synthesis^47^, and matrix metalloproteinase^48^, leading to liver fibrosis improvement.

US is an acceptable screening modality for MAFLD diagnosis. The echogenicity of the liver parenchyma increases with hepatocyte fat deposition. Based on ultrasonographic appearance, the grading of hepatic steatosis can be classified as mild, moderate, and severe. The RRs associated with the resolution of hepatic steatosis were pooled, and to the best of our knowledge, this is the first meta-analysis to consider these outcomes. Curcumin supplementation yielded approximately a 3.5-fold higher resolution rate of hepatic steatosis than placebo. It is widely accepted that MAFLD is strongly related to obesity and weight loss therapy may significantly improve liver steatosis^49–51^. This improvement in steatosis could therefore be mediated through weight loss associated with curcumin supplementation, although the efficacy of curcumin on weight reduction remains controversial^16,21,52–55^. In our updated review, significant weight loss was observed in some RCTs^23,25,34^ but not in the others^24,29,31,32,36,37^.

In this meta-analysis, improvement of liver fibrosis was assessed by transient elastography (FibroScan^®^) and no significant differences between curcumin and placebo were identified, although we were unable to pool this outcome given only a single RCT reported this outcome^31^.

In a curcumin formulation subgroup analysis, the effects on AST, ALT, and the resolution of hepatic steatosis remained significant in patients receiving bioavailability-enhanced forms, suggesting this curcumin supplement formulation may be more efficacious than crude extracts or turmeric powder. This is consistent with the fact that curcumin has very low water solubility leading to low absorption, as well as rapid metabolism^56,57^. As a consequence, curcumin bioavailability in the blood circulation is low. Phytosomal curcumin (i.e., curcumin-phospholipid complex), nano-micelles containing curcumin, amorphous solid dispersion of curcumin, and curcumin combined with piperine as an adjuvant represent curcumin formulations designed to improve gastrointestinal absorption, improving bioavailability and therapeutic effect^56,57^. However, non-significant associations with whole compounds may have resulted from inadequate power in studies with insufficient participant numbers.

It should be noted that curcumin might improve liver-specific outcomes in MAFLD patients through reducing some metabolic dysfunctions as reported in previous MAs suggesting improvement not only of lipid profile and weight control but also of glycemic outcomes after curcumin supplements^16,21^; corresponding to our findings which showed significant effect of curcumin on the improvement of FBS level. Our MA also indicated significant improvement of BMI in MAFLD patients receiving curcumin, and agreed with the recently published MA^58^ which also reported significant body weight and waist circumference reduction following curcumin supplement. Given that weight loss is the most effective treatment for steatosis and NASH^59^, curcumin supplement could be considered in MAFLD patients with obesity. Significant reduction in TC levels was observed from our evidence synthesis but not for LDL-C, HDL-C, and TG. Most included RCTs assessed curcumin supplementation for only 8 to 12 weeks; therefore, longer curcumin supplementation might be required to see the effects of curcumin on LDL-C, HDL-C, and TG levels. Further large-scale studies are required to prove if curcumin has a direct effect on MAFLD or its effect is through these metabolic and/or anthropometric measures. Recently, the relationship between MAFLD and metabolic syndrome have been suggested to be bidirectional^60–62^. In other words, treatments that alleviate MAFLD, such as curcumin, possibly work through well control of metabolic syndrome, or vice versa. From our findings, curcumin could improve multiple metabolic outcomes, which should be beneficial for MAFLD patients as for the current practice guideline^63^ that recommended intensive control of metabolic syndrome in MAFLD patients.

While life-style modification targeting weight loss remains the mainstay of MAFLD treatment, several medications have been proposed to improve MAFLD/NASH associated outcomes. According to treatment guidelines^63–65^, potential medications include insulin sensitizers (i.e., metformin and thiazolidinediones), glucagon-like peptide-1 agonists, vitamin E, ursodeoxycholic acid, and omega-3 fatty acid, etc. Of these, only pioglitazone, semaglutide, and vitamin E could improve steatosis or NASH^63–65^. Our study suggests that curcumin could be a nutraceutical option in the treatment of MAFLD patients. Curcumin safety was demonstrated in several RCTs with no major toxicity observed^66^. The major adverse reaction was gastrointestinal disturbance (i.e., nausea and diarrhea). However, curcumin may potentially interact with some medications given its inhibitory effect on cytochrome P450^67,68^. As such, appropriate consideration is required for prescription in patients with multiple comorbidities.

Our study had some limitations. First, most of the studies included were conducted in Iran, which might affect the generalizability of our findings. Second, several outcomes (i.e., GGT, hepatic steatosis improvement, and liver stiffness) were reported in only a small number of studies which adversely impacts the precision of the effect sizes reported. Third, various formulations of curcumin were used in different studies with the majority of RCTs using bioavailability-enhanced curcumin in contrast to several RCTs that used whole turmeric compounds or curcumin extract. As a consequence, the pooled results based on studies that used whole compounds or extracts may be less precise. Finally, the high levels of heterogeneity detected may be due to the variation in turmeric source and quality, extraction methods, and dosage between studies, which may reduce the robustness of the evidence synthesized.

Nevertheless, our comprehensive review provides support for the positive effects of curcumin on slowing the progression of MAFLD. We found a 3.5-fold significantly higher chance of resolution in hepatic steatosis, and a small but significant lowering in aminotransferase levels, FBS, BMI, and TC. However, improvements associated with NASH histology and liver fibrosis has not been confirmed in this review. Further trials should address these shortcomings given the small number of RCTs available to date.

## Supplementary Information

Supplementary Information.

## Data Availability

All data generated or analysed during this study are included in this published article and its supplementary information files.

## References

[1] Kim D, Touros A, Kim WR (2018). Nonalcoholic fatty liver disease and metabolic syndrome. Clin. Liver Dis..

[2] Younossi ZM, Henry L, Bush H, Mishra A (2018). Clinical and economic burden of nonalcoholic fatty liver disease and nonalcoholic steatohepatitis. Clin. Liver Dis..

[3] Golabi P (2021). Burden of non-alcoholic fatty liver disease in Asia, the Middle East and North Africa: Data from Global Burden of Disease 2009–2019. J. Hepatol..

[4] Lindenmeyer CC, McCullough AJ (2018). The natural history of nonalcoholic fatty liver disease—An evolving view. Clin. Liver Dis..

[5] Eslam M (2020). A new definition for metabolic dysfunction-associated fatty liver disease: An international expert consensus statement. J. Hepatol..

[6] Eslam M, Sanyal AJ, George J (2020). MAFLD: A consensus-driven proposed nomenclature for metabolic associated fatty liver disease. Gastroenterology.

[7] Alharthi J, Gastaldelli A, Cua IH, Ghazinian H, Eslam M (2022). Metabolic dysfunction-associated fatty liver disease: A year in review. Curr. Opin. Gastroenterol..

[8] Kuchay MS, Choudhary NS, Mishra SK (2020). Pathophysiological mechanisms underlying MAFLD. Diabetes Metab. Syndr..

[9] Eslam M (2020). The Asian Pacific Association for the Study of the Liver clinical practice guidelines for the diagnosis and management of metabolic associated fatty liver disease. Hepatol. Int..

[10] Prikhodko VA, Bezborodkina NN, Okovityi SV (2022). Pharmacotherapy for non-alcoholic fatty liver disease: Emerging targets and drug candidates. Biomedicines..

[11] Abenavoli L (2021). Dietary polyphenols and non-alcoholic fatty liver disease. Nutrients.

[12] Jabczyk M, Nowak J, Hudzik B, Zubelewicz-Szkodzińska B (2021). Curcumin in metabolic health and disease. Nutrients.

[13] Goodarzi R, Sabzian K, Shishehbor F, Mansoori A (2019). Does turmeric/curcumin supplementation improve serum alanine aminotransferase and aspartate aminotransferase levels in patients with nonalcoholic fatty liver disease? A systematic review and meta-analysis of randomized controlled trials. Phytother. Res..

[14] Mansour-Ghanaei F, Pourmasoumi M, Hadi A, Joukar F (2019). Efficacy of curcumin/turmeric on liver enzymes in patients with non-alcoholic fatty liver disease: A systematic review of randomized controlled trials. Integr. Med. Res..

[15] Wei Z (2019). The effects of curcumin on the metabolic parameters of non-alcoholic fatty liver disease: A meta-analysis of randomized controlled trials. Hepatol. Int..

[16] Jalali M (2020). The effects of curcumin supplementation on liver function, metabolic profile and body composition in patients with non-alcoholic fatty liver disease: A systematic review and meta-analysis of randomized controlled trials. Complement. Ther. Med..

[17] Zhou J (2021). The efficacy of novel metabolic targeted agents and natural plant drugs for nonalcoholic fatty liver disease treatment: A PRISMA-compliant network meta-analysis of randomized controlled trials. Medicine (Baltimore).

[18] Hutton B (2015). The PRISMA extension statement for reporting of systematic reviews incorporating network meta-analyses of health care interventions: Checklist and explanations. Ann. Intern. Med..

[19] Whiting P (2016). ROBIS: A new tool to assess risk of bias in systematic reviews was developed. J. Clin. Epidemiol..

[20] Sterne JAC (2019). RoB 2: A revised tool for assessing risk of bias in randomised trials. BMJ.

[21] Khalili L, Nammi S (2022). The effects of curcumin supplementation on metabolic biomarkers and body mass index in patients with nonalcoholic fatty liver disease, a systematic review and meta-analysis of randomized controlled trials. Curr. Pharm. Des..

[22] Chirapongsathorn S (2012). Curcumin trend to improve alanine transaminase (ALT) in non-alcoholic fatty liver disease (NAFLD) with abnormal ALT. Integr. Med. Res..

[23] Rahmani S (2016). Treatment of non-alcoholic fatty liver disease with curcumin: A randomized placebo-controlled trial. Phytother. Res..

[24] Navekar R, Rafraf M, Ghaffari A, Asghari-Jafarabadi M, Khoshbaten M (2017). Turmeric supplementation improves serum glucose indices and leptin levels in patients with nonalcoholic fatty liver diseases. J. Am. Coll. Nutr..

[25] Panahi Y (2017). Efficacy and safety of phytosomal curcumin in non-alcoholic fatty liver disease: A randomized controlled trial. Drug Res. (Stuttg)..

[26] Saadati S (2018). Comparing different non-invasive methods in assessment of the effects of curcumin on hepatic fibrosis in patients with non-alcoholic fatty liver disease. Gastroenterol. Hepatol. Bed Bench..

[27] Chashmniam S (2019). A pilot study of the effect of phospholipid curcumin on serum metabolomic profile in patients with non-alcoholic fatty liver disease: A randomized, double-blind, placebo-controlled trial. Eur. J. Clin. Nutr..

[28] Jazayeri-Tehrani SA (2019). Nano-curcumin improves glucose indices, lipids, inflammation, and Nesfatin in overweight and obese patients with non-alcoholic fatty liver disease (NAFLD): A double-blind randomized placebo-controlled clinical trial. Nutr. Metab. (Lond.).

[29] Mirhafez SR (2019). Effect of phytosomal curcumin on circulating levels of adiponectin and leptin in patients with non-alcoholic fatty liver disease: A randomized, double-blind, placebo-controlled clinical trial. J. Gastrointest. Liver Dis..

[30] Panahi Y (2019). Curcuminoids plus piperine improve nonalcoholic fatty liver disease: A clinical trial. J. Cell Biochem..

[31] Saadati S (2019). The effects of curcumin supplementation on liver enzymes, lipid profile, glucose homeostasis, and hepatic steatosis and fibrosis in patients with non-alcoholic fatty liver disease. Eur. J. Clin. Nutr..

[32] Hariri M, Gholami A, Mirhafez SR, Bidkhori M, Sahebkar A (2020). A pilot study of the effect of curcumin on epigenetic changes and DNA damage among patients with non-alcoholic fatty liver disease: A randomized, double-blind, placebo-controlled, clinical trial. Complement. Ther. Med..

[33] Moradi Kelardeh B, Rahmati-Ahmadabad S, Farzanegi P, Helalizadeh M, Azarbayjani MA (2020). Effects of non-linear resistance training and curcumin supplementation on the liver biochemical markers levels and structure in older women with non-alcoholic fatty liver disease. J. Bodyw. Mov. Ther..

[34] Saberi-Karimian M (2020). Effects of curcuminoids on inflammatory status in patients with non-alcoholic fatty liver disease: A randomized controlled trial. Complement. Ther. Med..

[35] Jarhahzadeh M, Alavinejad P, Farsi F, Husain D, Rezazadeh A (2021). The effect of turmeric on lipid profile, malondialdehyde, liver echogenicity and enzymes among patients with nonalcoholic fatty liver disease: A randomized double blind clinical trial. Diabetol. Metab. Syndr..

[36] Mirhafez SR (2021). The effect of curcumin phytosome on the treatment of patients with non-alcoholic fatty liver disease: A double-blind, randomized, placebo-controlled trial. Adv. Exp. Med. Biol..

[37] Mirhafez SR (2021). Curcumin and piperine combination for the treatment of patients with non-alcoholic fatty liver disease: A double-blind randomized placebo-controlled trial. Adv. Exp. Med. Biol..

[38] Cicero AFG (2020). Effects of phytosomal curcumin on anthropometric parameters, insulin resistance, cortisolemia and non-alcoholic fatty liver disease indices: A double-blind, placebo-controlled clinical trial. Eur. J. Nutr..

[39] Nouri-Vaskeh M, Malek Mahdavi A, Afshan H, Alizadeh L, Zarei M (2020). Effect of curcumin supplementation on disease severity in patients with liver cirrhosis: A randomized controlled trial. Phytother. Res..

[40] Farzaei MH (2018). Curcumin in liver diseases: A systematic review of the cellular mechanisms of oxidative stress and clinical perspective. Nutrients.

[41] Gao TH (2022). *Curcumae*
*rhizoma* and its major constituents against hepatobiliary disease: Pharmacotherapeutic properties and potential clinical applications. Phytomedicine.

[42] Piper JT (1998). Mechanisms of anticarcinogenic properties of curcumin: The effect of curcumin on glutathione linked detoxification enzymes in rat liver. Int. J. Biochem. Cell Biol..

[43] Motterlini R, Foresti R, Bassi R, Green CJ (2000). Curcumin, an antioxidant and anti-inflammatory agent, induces heme oxygenase-1 and protects endothelial cells against oxidative stress. Free Radic. Biol. Med..

[44] Mohammadi S, KarimzadehBardei L, Hojati V, Ghorbani AG, Nabiuni M (2017). Anti-inflammatory effects of curcumin on insulin resistance index, levels of interleukin-6, C-reactive protein, and liver histology in polycystic ovary syndrome-induced rats. Cell J..

[45] Ho WI (2022). Liposome-encapsulated curcumin attenuates HMGB1-mediated hepatic inflammation and fibrosis in a murine model of Wilson's disease. Biomed. Pharmacother..

[46] Damiano S (2021). Antioxidative effects of curcumin on the hepatotoxicity induced by Ochratoxin A in rats. Antioxidants (Basel).

[47] Kang HC (2002). Curcumin inhibits collagen synthesis and hepatic stellate cell activation in-vivo and in-vitro. J. Pharm. Pharmacol..

[48] Rajagopalan R, Sridharana S, Menon VP (2010). Hepatoprotective role of bis-demethoxy curcumin analog on the expression of matrix metalloproteinase induced by alcohol and polyunsaturated fatty acid in rats. Toxicol. Mech. Methods.

[49] Milić S, Lulić D, Štimac D (2014). Non-alcoholic fatty liver disease and obesity: Biochemical, metabolic and clinical presentations. World J. Gastroenterol..

[50] Polyzos SA, Kountouras J, Mantzoros CS (2019). Obesity and nonalcoholic fatty liver disease: From pathophysiology to therapeutics. Metabolism.

[51] Hashem A, Khalouf A, Acosta A (2021). Management of obesity and nonalcoholic fatty liver disease: A literature review. Semin. Liver Dis..

[52] Akbari M (2019). The effects of curcumin on weight loss among patients with metabolic syndrome and related disorders: A systematic review and meta-analysis of randomized controlled trials. Front. Pharmacol..

[53] Jafarirad S (2019). Does turmeric/curcumin supplementation change anthropometric indices in patients with non-alcoholic fatty liver disease? A systematic review and meta-analysis of randomized controlled trials. Clin. Nutr. Res..

[54] Baziar N, Parohan M (2020). The effects of curcumin supplementation on body mass index, body weight, and waist circumference in patients with nonalcoholic fatty liver disease: A systematic review and dose-response meta-analysis of randomized controlled trials. Phytother. Res..

[55] Mousavi SM, Milajerdi A, Varkaneh HK, Gorjipour MM, Esmaillzadeh A (2020). The effects of curcumin supplementation on body weight, body mass index and waist circumference: A systematic review and dose-response meta-analysis of randomized controlled trials. Crit. Rev. Food Sci. Nutr..

[56] Mirzaei H (2017). Phytosomal curcumin: A review of pharmacokinetic, experimental and clinical studies. Biomed. Pharmacother..

[57] Stohs SJ (2020). Highly bioavailable forms of curcumin and promising avenues for curcumin-based research and application: A review. Molecules.

[58] Unhapipatpong, C. *et al.* The effect of curcumin supplementation on weight loss and anthropometric indices: An umbrella review and updated meta-analyses of randomized controlled trials. *Am. J. Clin. Nutr.* (2023). 10.1016/j.ajcnut.2023.03.006 36898635

[59] Finer N (2022). Weight loss interventions and nonalcoholic fatty liver disease: Optimizing liver outcomes. Diabetes Obes. Metab..

[60] Lonardo A, Nascimbeni F, Mantovani A, Targher G (2018). Hypertension, diabetes, atherosclerosis and NASH: Cause or consequence?. J. Hepatol..

[61] Morrison AE, Zaccardi F, Khunti K, Davies MJ (2019). Causality between non-alcoholic fatty liver disease and risk of cardiovascular disease and type 2 diabetes: A meta-analysis with bias analysis. Liver Int..

[62] Targher G, Corey KE, Byrne CD, Roden M (2021). The complex link between NAFLD and type 2 diabetes mellitus—Mechanisms and treatments. Nat. Rev. Gastroenterol. Hepatol..

[63] Rinella, M. E. *et al.* AASLD Practice Guidance on the clinical assessment and management of nonalcoholic fatty liver disease. *Hepatology* (2023). 10.1097/HEP.0000000000000323 PMC10735173 36727674

[64] EASL-EASD-EASO Clinical Practice Guidelines for the management of non-alcoholic fatty liver disease. *J. Hepatol*. **64**, 1388–1402 (2016). 10.1016/j.jhep.2015.11.004 27062661

[65] Chalasani N (2018). The diagnosis and management of nonalcoholic fatty liver disease: Practice guidance from the American Association for the Study of Liver Diseases. Hepatology.

[66] Soleimani V, Sahebkar A, Hosseinzadeh H (2018). Turmeric (*Curcuma longa*) and its major constituent (curcumin) as nontoxic and safe substances: Review. Phytother. Res..

[67] Volak LP, Ghirmai S, Cashman JR, Court MH (2008). Curcuminoids inhibit multiple human cytochromes P450, UDP-glucuronosyltransferase, and sulfotransferase enzymes, whereas piperine is a relatively selective CYP3A4 inhibitor. Drug Metab. Dispos..

[68] Wang Z (2015). Inhibitory effects of curcumin on activity of cytochrome P450 2C9 enzyme in human and 2C11 in rat liver microsomes. Drug Dev. Ind. Pharm..

