# Supplementary material for: An updated meta-analysis of effects of curcumin on metabolic dysfunction-associated fatty liver disease based on available evidence from Iran and Thailand
Source: Sci Rep. 2023 Apr 10;13:5824. doi: 10.1038/s41598-023-33023-3 (PMC10086025; doi:10.1038/s41598-023-33023-3)
Supplement: Supplementary file 1 — Supplementary Information. [file 41598_2023_33023_MOESM1_ESM.docx]

Appendices

**An updated meta-analysis of effects of curcumin on metabolic dysfunction-associated fatty liver disease based on available evidence from Iran and Thailand**

1. Search terms
2. Assessment of the methodological quality using the ROBIS instrument for the systematic reviews included.
3. Study-citation matrix for aspartate aminotransferase and alanine aminotransferase outcomes
4. Forest plots of pooled effect sizes from included systematic review and meta-analyses: aspartate aminotransferase
5. Forest plots of pooled effect sizes from included systematic review and meta-analyses: alanine aminotransferase
6. Risk of bias summary assessment for randomized-controlled trials included within the updated meta-analysis
7. Risk of bias assessment for randomized-controlled trials included within the updated meta-analysis
8. Forest plots demonstrating the effects of curcumin on aspartate aminotransferase: bioavailability-enhanced form
9. Forest plots demonstrating the effects of curcumin on alanine aminotransferase: bioavailability-enhanced form
10. Forest plots of the effects of curcumin on alkaline phosphatase
11. Forest plots of the effects of curcumin on gamma-glutamyl transpeptidase
12. Forest plots demonstrating the effects of curcumin on the improvement of hepatic steatosis assessed by ultrasonography
13. Forest plots demonstrating the effects of curcumin on liver stiffness assessed by transient elastography
14. Forest plots demonstrating the effects of curcumin on platelet count
15. Forest plots demonstrating the effects of curcumin on fasting blood sugar
16. Forest plots demonstrating the effects of curcumin on HbA1c
17. Forest plots demonstrating the effects of curcumin on body mass index (BMI)
18. Forest plots demonstrating the effects of curcumin on total cholesterol (TC)
19. Forest plots demonstrating the effects of curcumin on LDL-C
20. Forest plots demonstrating the effects of curcumin on HDL-C
21. Forest plots demonstrating the effects of curcumin on triglycerides (TG)
22. Forest plots demonstrating the effects of curcumin on systolic blood pressure (SBP)
23. Forest plots demonstrating the effects of curcumin on diastolic blood pressure (DBP)
24. Sensitivity analysis by excluding studies with high risk of bias: aspartate aminotransferase
25. Sensitivity analysis by excluding studies with high risk of bias: alanine aminotransferase
26. Sensitivity analysis by excluding studies with high risk of bias: the resolution of hepatic steatosis
27. Funnel plots of aspartate aminotransferase
28. Funnel plots of alanine aminotransferase
29. Funnel plots of alkaline phosphatase
30. Funnel plots of the resolution of hepatic steatosis
31. Funnel plots of BMI
32. Funnel plots of TC
33. Funnel plots of TG
34. Funnel plots of the improvement of hepatic steatosis
35. Contour-enhanced funnel plots of the improvement of hepatic steatosis
36. Funnel plots of fasting blood sugar
37. Contour-enhanced funnel plots of fasting blood sugar
38. Funnel plots of HbA1c
39. Funnel plots of LDL-C
40. Contour-enhanced funnel plots of LDL-C
41. Funnel plots of HDL-C
42. Contour-enhanced funnel plots of HDL-C
43. Funnel plots of SBP
44. Contour-enhanced funnel plots of SBP
45. Funnel plots of DBP
46. Contour-enhanced funnel plots of DBP

Appendix A. Search terms

| **Domain** | **Search** | **Search Term** |
| --- | --- | --- |
| P | #1 | nonalcoholic |
|  | #2 | non-alcoholic |
|  | #3 | “non alcoholic” |
|  | #4 | “fatty liver disease” |
|  | #5 | Steatosis |
|  | #6 | NAFLD |
|  | #7 | “metabolic associated fatty liver disease” |
|  | #8 | MAFLD |
|  | #9 | steatohepatitis |
|  | #10 | NASH |
|  | #11 | ((nonalcoholic OR non-alcoholic OR “non alcoholic”) AND “fatty liver disease”)  OR (NAFLD OR “metabolic associated fatty liver disease” OR MAFLD OR steatosis OR steatohepatitis OR NASH) |
| I | #12 | Curcumin |
|  | #13 | “curcuma domestica” |
|  | #14 | “curcuma longa” |
|  | #15 | Turmeric |
|  | #16 | tumeric |
|  | #17 | curcuminoid |
|  | #18 | curcumin OR “curcuma domestica” OR “curcuma longa” OR turmeric OR tumeric OR curcuminoid |
| P & I | #19 | (((nonalcoholic OR non-alcoholic OR “non alcoholic”) AND “fatty liver disease”) OR (NAFLD OR “metabolic associated fatty liver disease” OR MAFLD OR steatosis OR steatohepatitis OR NASH))  AND (curcumin OR “curcuma domestica” OR “curcuma longa” OR turmeric OR tumeric OR curcuminoid) |

Appendix B. Assessment of the methodological quality using the ROBIS instrument for the systematic reviews included.

| **First author, year** | **1. STUDY ELIGIBILITY CRITERIA** | **2. IDENTIFICATION AND SELECTION OF STUDIES** | **3. DATA COLLECTION AND STUDY APPRAISAL** | **4. SYNTHESIS AND FINDINGS** | **RISK OF BIAS IN THE REVIEW** |
| --- | --- | --- | --- | --- | --- |
| Goodarzi, 2019 | ☺ | ☺ | ☺ | ☹ | ☺ |
| Mansour-Ghanaei, 2019 | ☺ | ☺ | ? | ☹ | ☹ |
| Wei, 2019 | ☺ | ☺ | ☺ | ☹ | ☺ |
| Jalali, 2020 | ☺ | ☹ | ? | ☹ | ☹ |
| Zhou, 2021 | ☺ | ☺ | ☹ | ☹ | ☹ |
| Khalili, 2022 | ☺ | ☺ | ☹ | ☹ | ☹ |

☺ = low risk; ☹ = high risk; and ? = unclear risk.

Appendix C. Study-citation matrix for aspartate aminotransferase and alanine aminotransferase outcomes

| First author, Year | | Systematic review and meta-analysis | | | | | | Total |
| --- | --- | --- | --- | --- | --- | --- | --- | --- |
|  |  | Goodazi,  2019 | Mansour-Ghanaei, 2019 | Wei, 2020 | Jalali, 2020 | Zhou, 2021 | Khalili, 2022 |  |
| Randomized controlled trial | Chirapongsathorn, 2012 | 1 | 0 | 1 | 0 | 0 | 0 | 2 |
|  | Moradi, 2016 | 1 | 1 | 0 | 0 | 0 | 0 | 2 |
|  | Panahi, 2016 | 0 | 1 | 0 | 0 | 0 | 0 | 1 |
|  | Rahmani, 2016 | 1 | 1 | 1 | 1 | 1 | 1 | 6 |
|  | Navekar, 2017 | 1 | 1 | 0 | 0 | 1 | 0 | 3 |
|  | Panahi, 2017 | 1 | 0 | 0 | 1 | 1 | 0 | 3 |
|  | Sadaati, 2018 | 0 | 0 | 0 | 0 | 0 | 1 | 1 |
|  | Chashmniam, 2019 | 0 | 0 | 0 | 1 | 1 | 0 | 2 |
|  | Panahi, 2019 | 0 | 0 | 0 | 0 | 1 | 0 | 1 |
|  | Jazayeri-Tehrani, 2019 | 0 | 0 | 0 | 1 | 1 | 1 | 3 |
|  | Mirhafez, 2019a | 0 | 0 | 0 | 1 | 1 | 1 | 3 |
|  | Saadati, 2019a | 0 | 0 | 0 | 1 | 0 | 1 | 2 |
|  | Saadati, 2019b | 0 | 0 | 0 | 1 | 1 | 1 | 3 |
|  | Cicero, 2020 | 0 | 0 | 0 | 0 | 1 | 1 | 2 |
|  | Hariri, 2020 | 0 | 0 | 0 | 0 | 1 | 1 | 2 |
|  | Husain, 2020 | 0 | 0 | 0 | 0 | 0 | 1 | 1 |
|  | Moradi, 2020 | 0 | 0 | 0 | 0 | 1 | 1 | 2 |
|  | Nouri-Vaskeh, 2020 | 0 | 0 | 0 | 0 | 0 | 1 | 1 |
|  | Saberi-Karimian, 2020 | 0 | 0 | 0 | 0 | 1 | 1 | 2 |
|  | Jarhahzadeh, 2021 | 1 | 0 | 0 | 0 | 0 | 0 | 1 |
|  | Mirhafez, 2021b | 0 | 0 | 0 | 0 | 1 | 0 | 1 |
| Total | | 6 | 4 | 2 | 7 | 13 | 12 | 44 |

Appendix D. Forest plots of pooled effect sizes from included systematic review and meta-analyses: aspartate aminotransferase


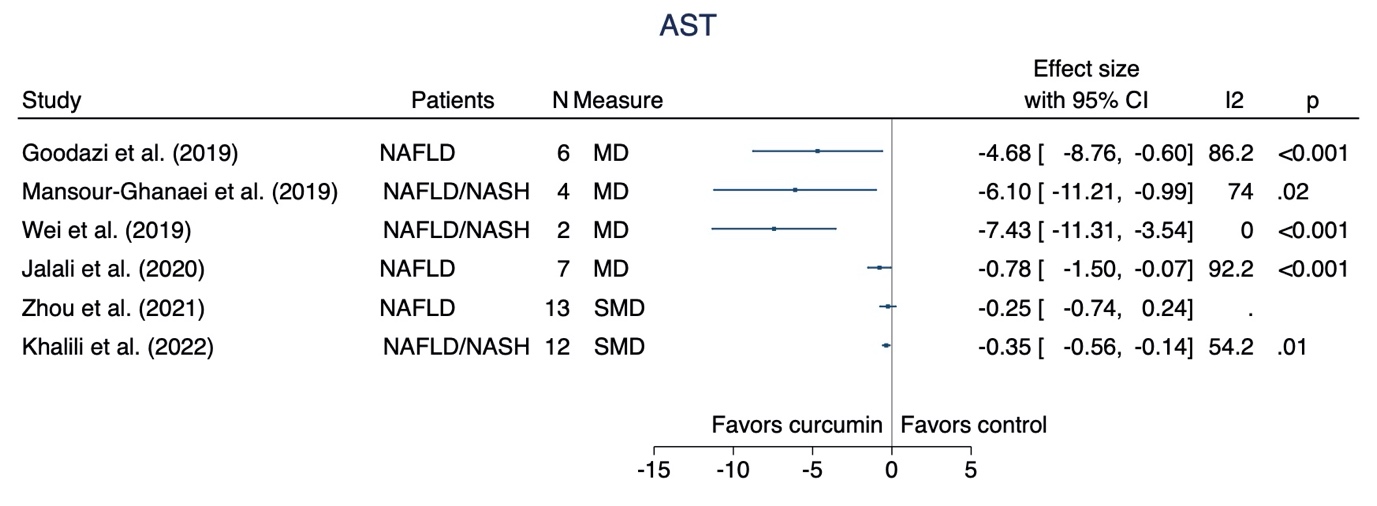


Appendix E. Forest plots of pooled effect sizes from included systematic review and meta-analyses: alanine aminotransferase


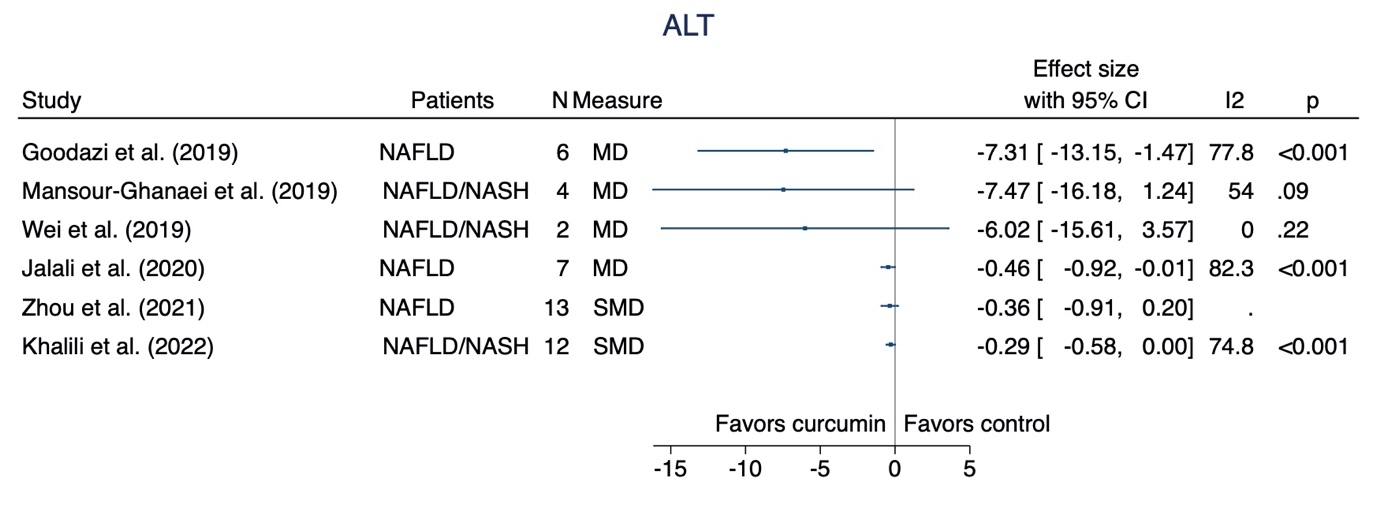


Appendix F. Risk of bias summary assessment for randomized-controlled trials included within the updated meta-analysis


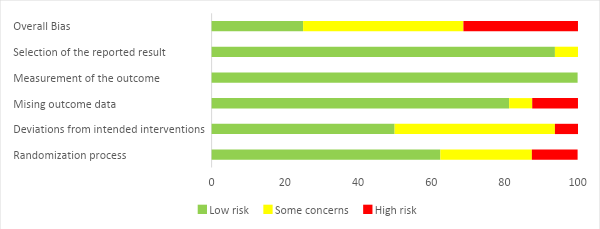


Appendix G. Risk of bias assessment for randomized-controlled trials included within the updated meta-analysis

| Study | Randomization | Protocol deviation | Missing outcome | Outcome measurement | Reporting | Overall |
| --- | --- | --- | --- | --- | --- | --- |
| Chirapongsathorn 2012 | S | H | L | L | S | H |
| Rahmani 2016 | S | S | L | L | L | S |
| Navekar 2017 | L | S | L | L | L | S |
| Panahi 2017 | L | S | S | L | L | S |
| Saadati 2018 | L | L | L | L | L | L |
| Chashmniam 2019 | S | L | L | L | L | S |
| Jazayeri-Tehrani 2019 | L | L | L | L | L | L |
| Mirhafez 2019a | H | L | L | L | L | H |
| Panahi 2019 | H | L | L | L | L | H |
| Saadati 2019a | L | L | L | L | L | L |
| Hariri 2020 | L | S | L | L | L | S |
| Moradi-Kelardeh 2020 | L | L | L | L | L | L |
| Saberi-Karimian 2020 | L | S | L | L | L | S |
| Jarhahzadeh 2021 | S | L | L | L | L | S |
| Mirhafez 2021a | L | S | H | L | L | H |
| Mirhafez 2021c | L | S | H | L | L | H |

H = high; L = low; S = some concerns

Appendix H. Forest plots demonstrating the effects of curcumin on aspartate aminotransferase: bioavailability-enhanced form

Appendix I. Forest plots demonstrating the effects of curcumin on alanine aminotransferase: bioavailability-enhanced form

Appendix J. Forest plots of the effects of curcumin on alkaline phosphatase


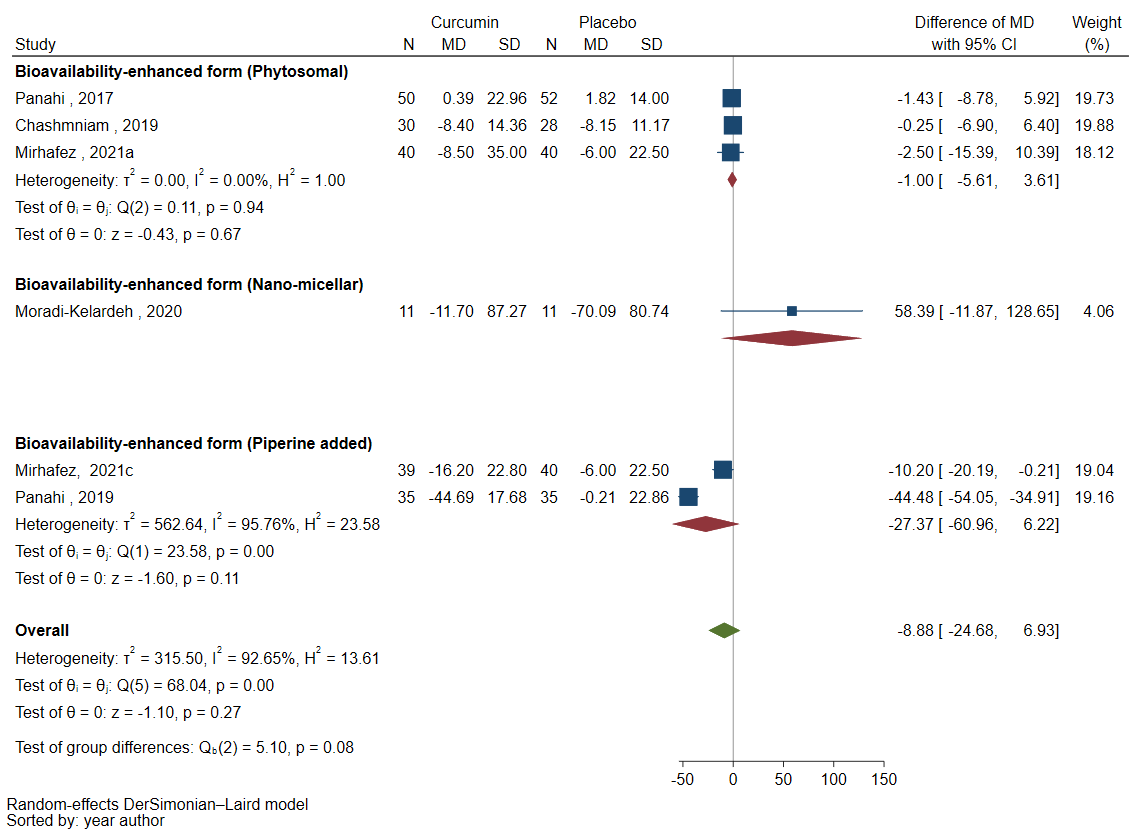


Appendix K. Forest plots of the effects of curcumin on gamma-glutamyl transpeptidase


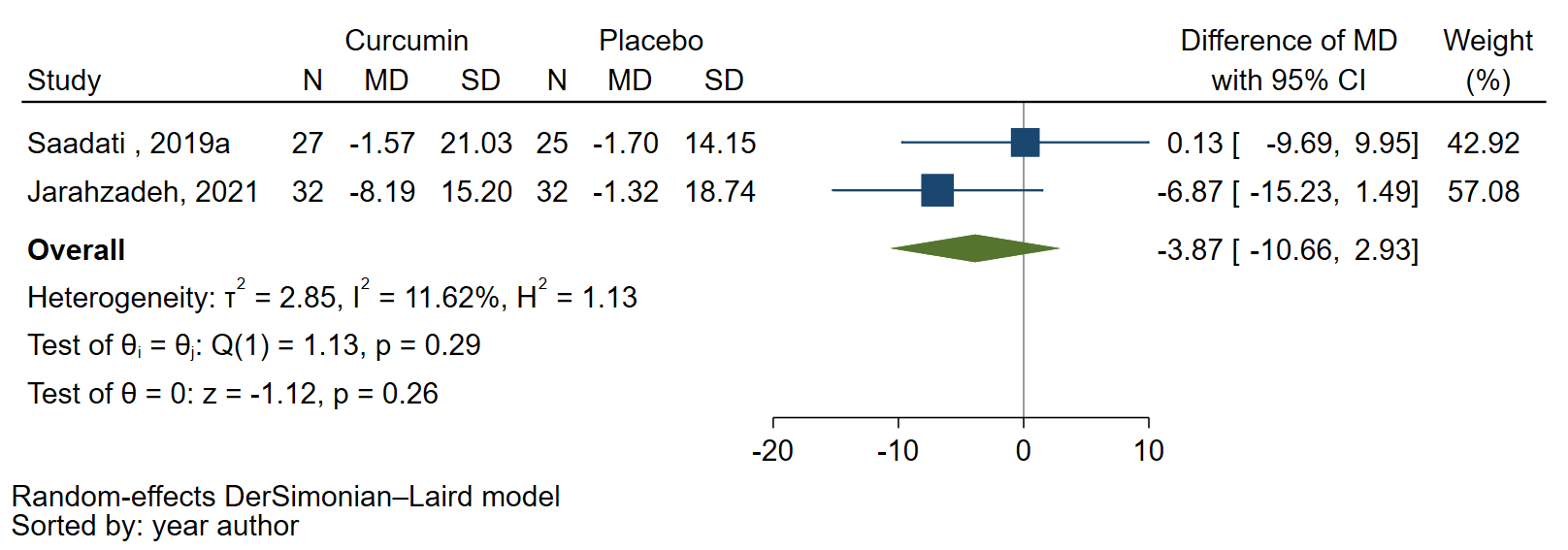


Appendix L. Forest plots demonstrating the effects of curcumin on the improvement of hepatic steatosis assessed by ultrasonography

Appendix M. Forest plots demonstrating the effects of curcumin on liver stiffness assessed by transient elastography

Appendix N. Forest plots demonstrating the effects of curcumin on platelet count

Appendix O. Forest plots demonstrating the effects of curcumin on reduction of fasting blood sugar after receiving curcumin supplementation


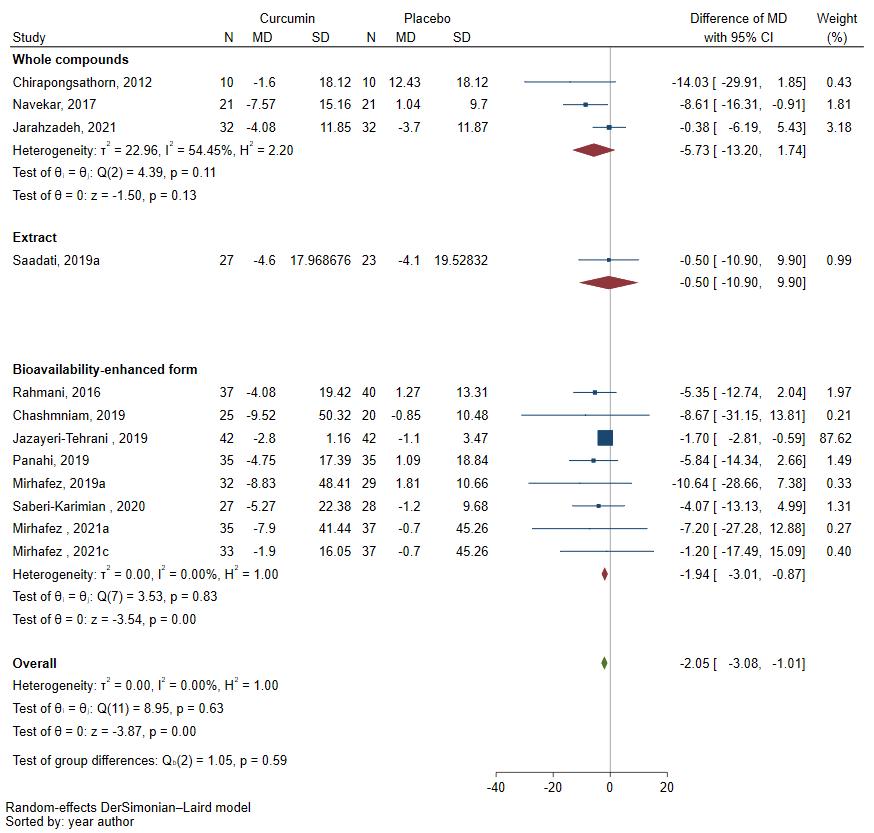


Appendix P. Forest plots demonstrating the effects of curcumin on reduction of HbA1c


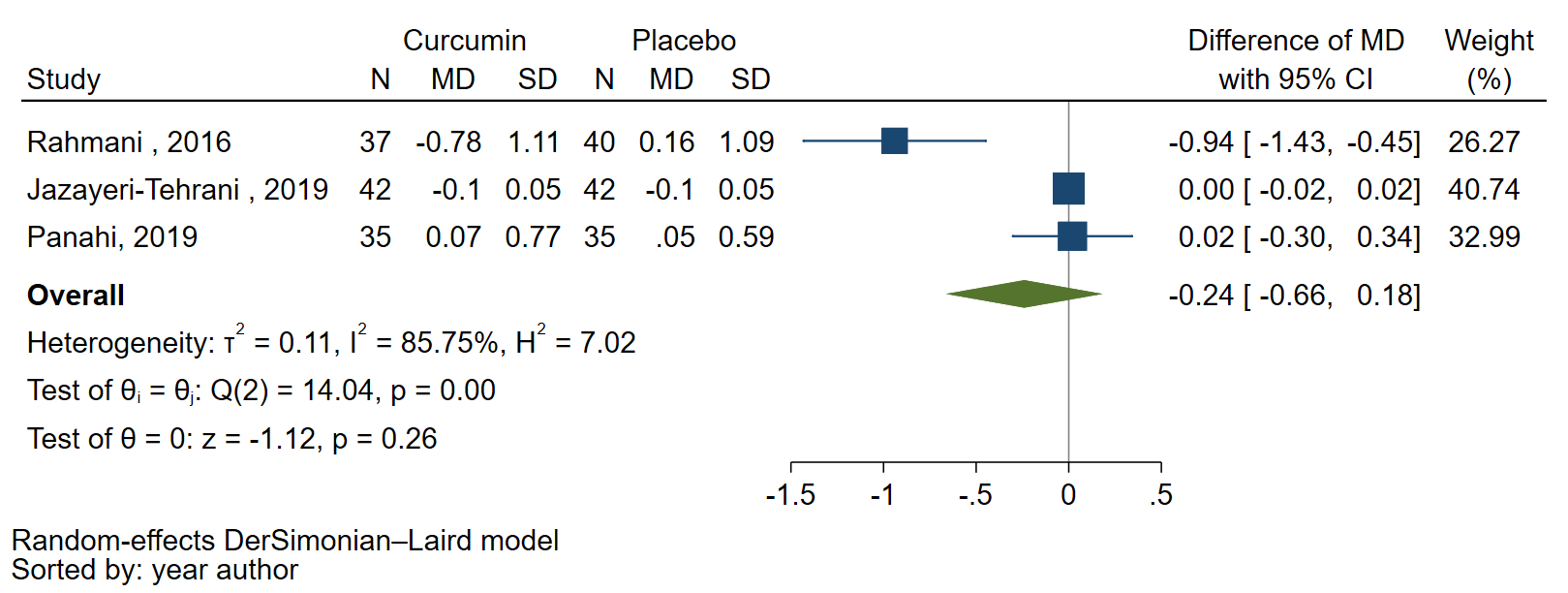


Appendix Q. Forest plots demonstrating the effects of curcumin on body mass index (BMI)

Appendix R. Forest plots demonstrating the effects of curcumin on total cholesterol (TC)

Appendix S. Forest plots demonstrating the effects of curcumin on LDL-C

Appendix T. Forest plots demonstrating the effects of curcumin on HDL-C

Appendix U. Forest plots demonstrating the effects of curcumin on triglycerides (TG)

Appendix V. Forest plots demonstrating the effects of curcumin on systolic blood pressure (SBP)

Appendix W. Forest plots demonstrating the effects of curcumin on diastolic blood pressure (DBP)

Appendix X. Sensitivity analysis by excluding studies with high risk of bias: aspartate aminotransferase


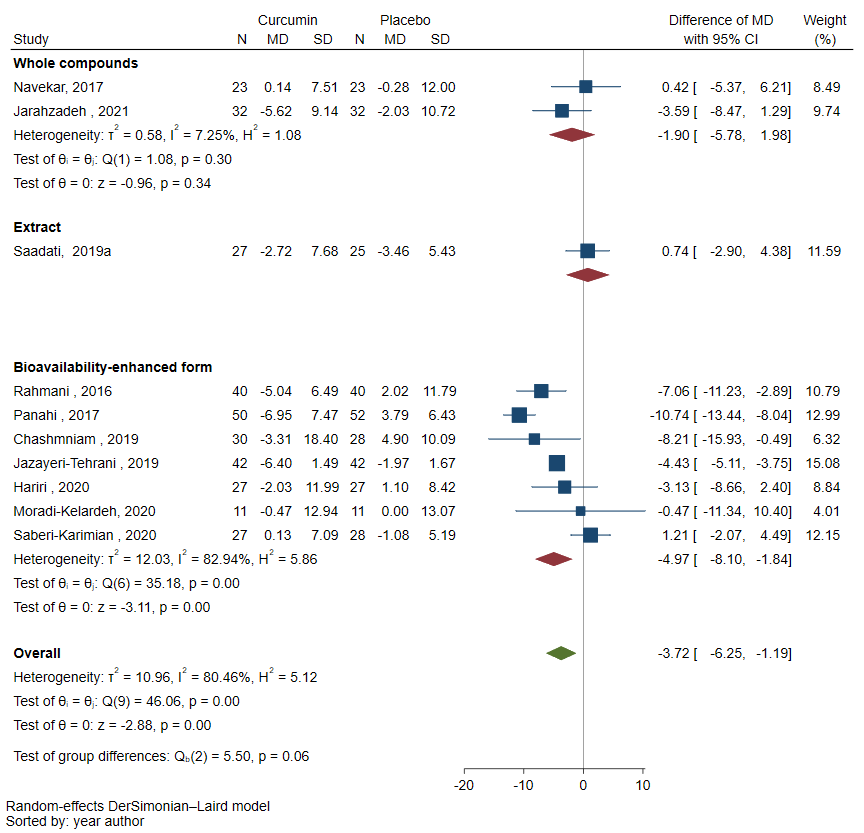


Appendix Y. Sensitivity analysis by excluding studies with high risk of bias: alanine aminotransferase


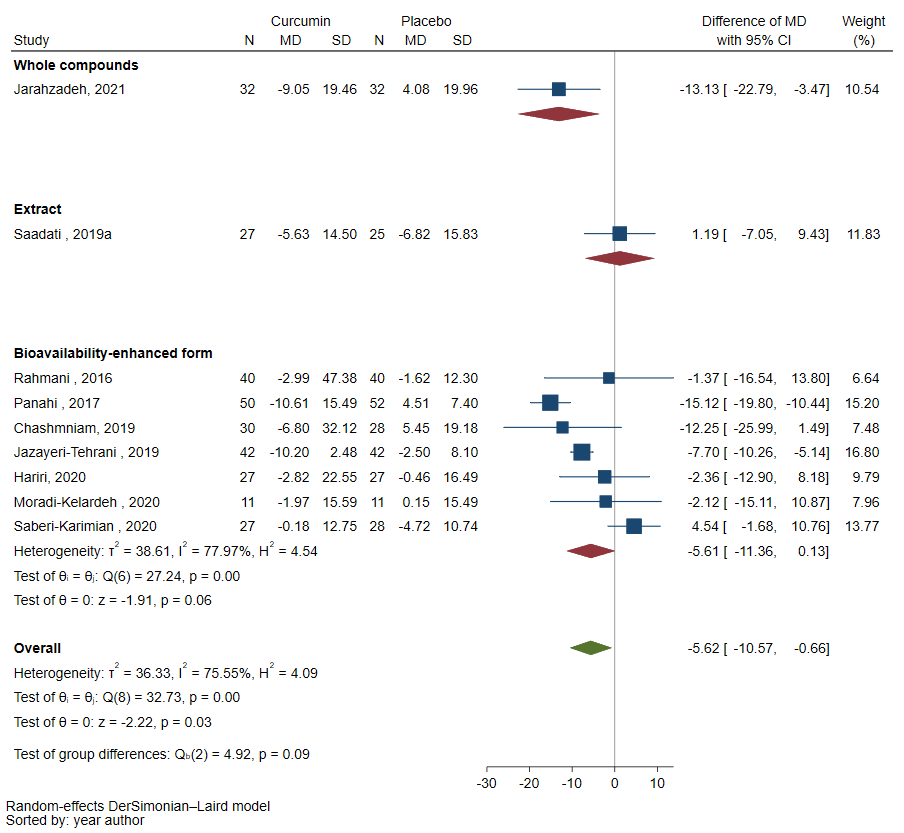


Appendix Z. Sensitivity analysis by excluding studies with high risk of bias: the resolution of hepatic steatosis


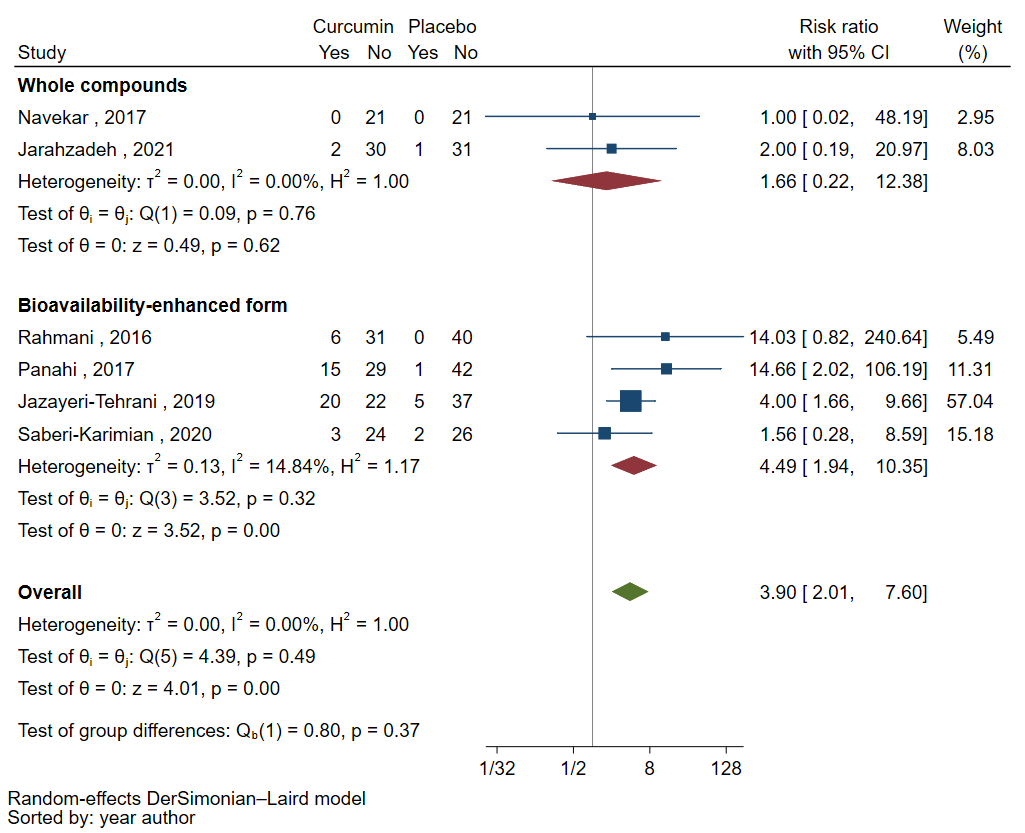


Appendix AA. Funnel plots of aspartate aminotransferase


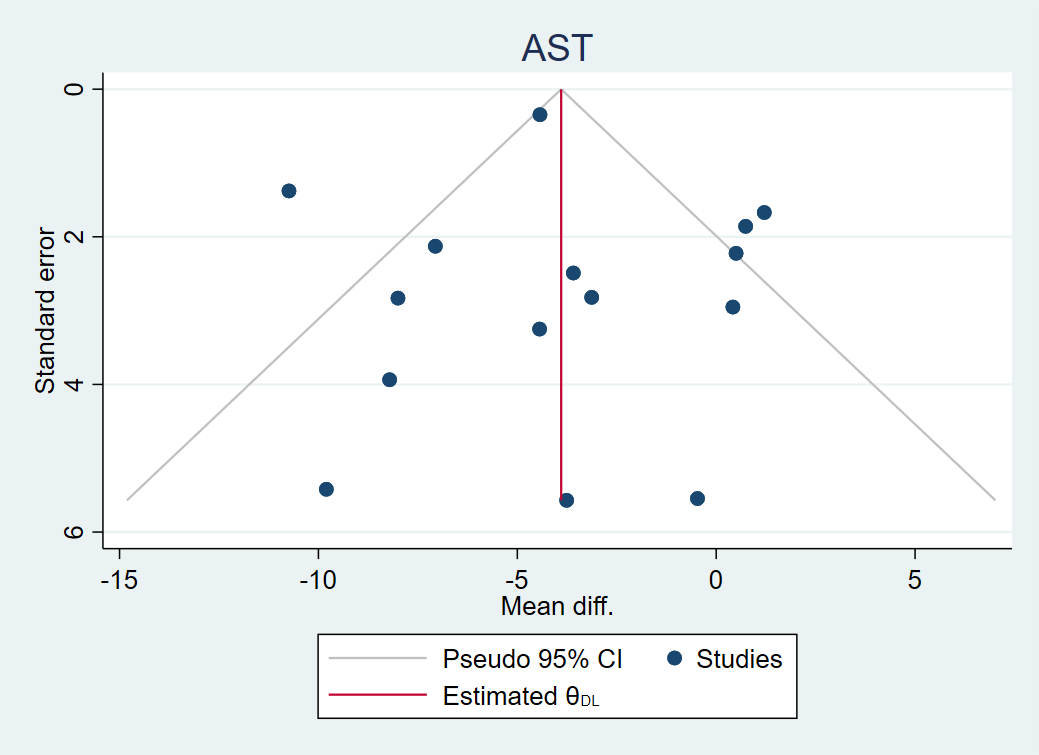


Appendix BB. Funnel plots of alanine aminotransferase


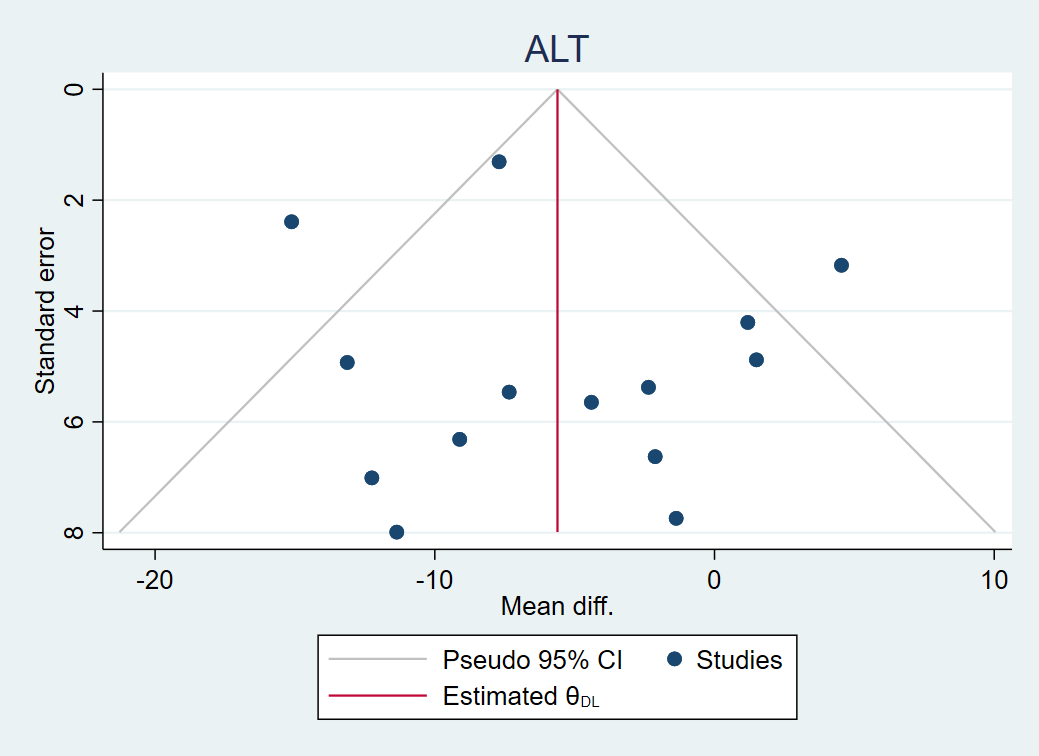


Appendix CC. Funnel plots of alkaline phosphatase


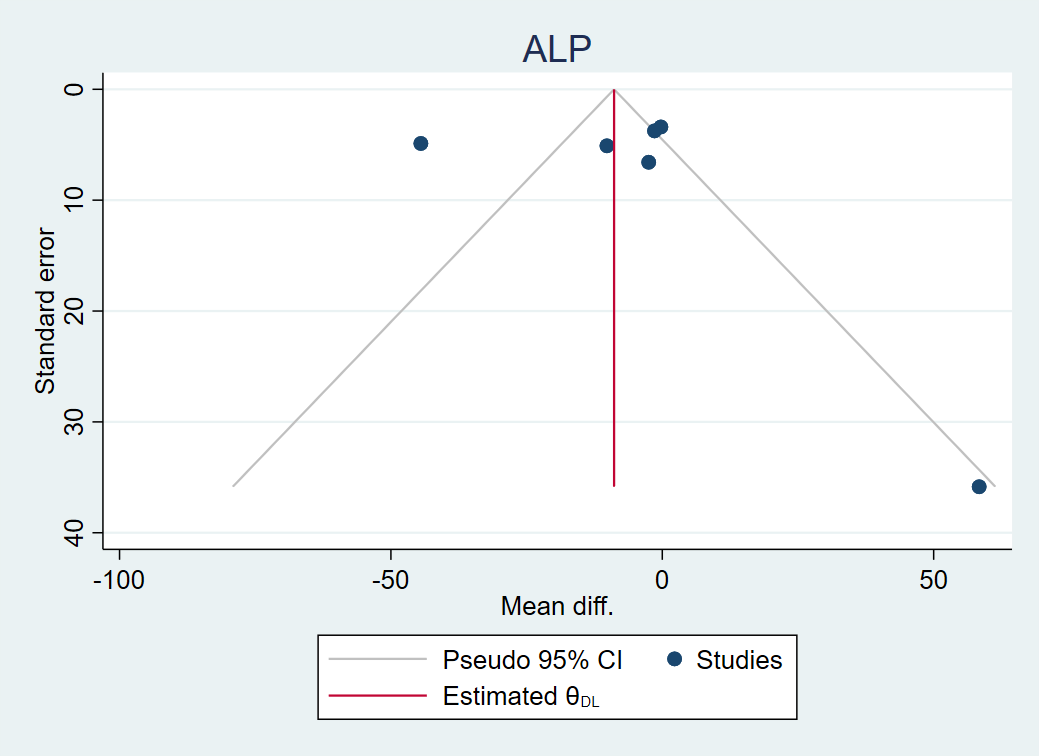


Appendix DD. Funnel plots of the resolution of hepatic steatosis


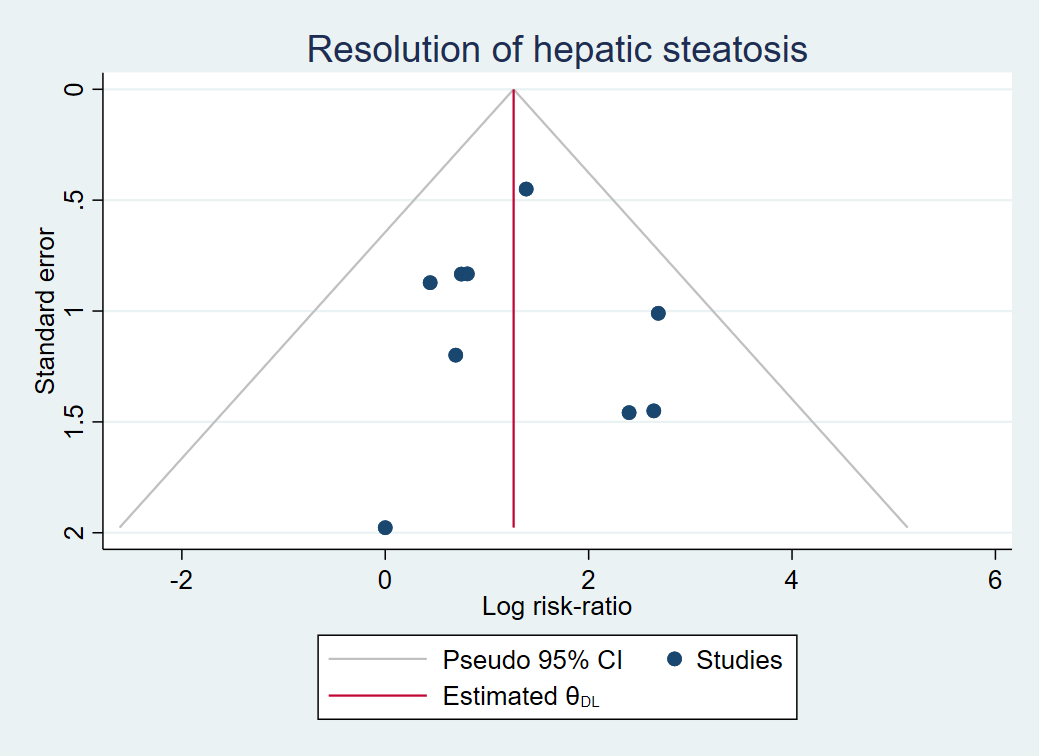


Appendix EE. Funnel plots of BMI

Appendix FF. Funnel plots of TC

Appendix GG. Funnel plots of TG

Appendix HH. Funnel plots of the improvement of hepatic steatosis


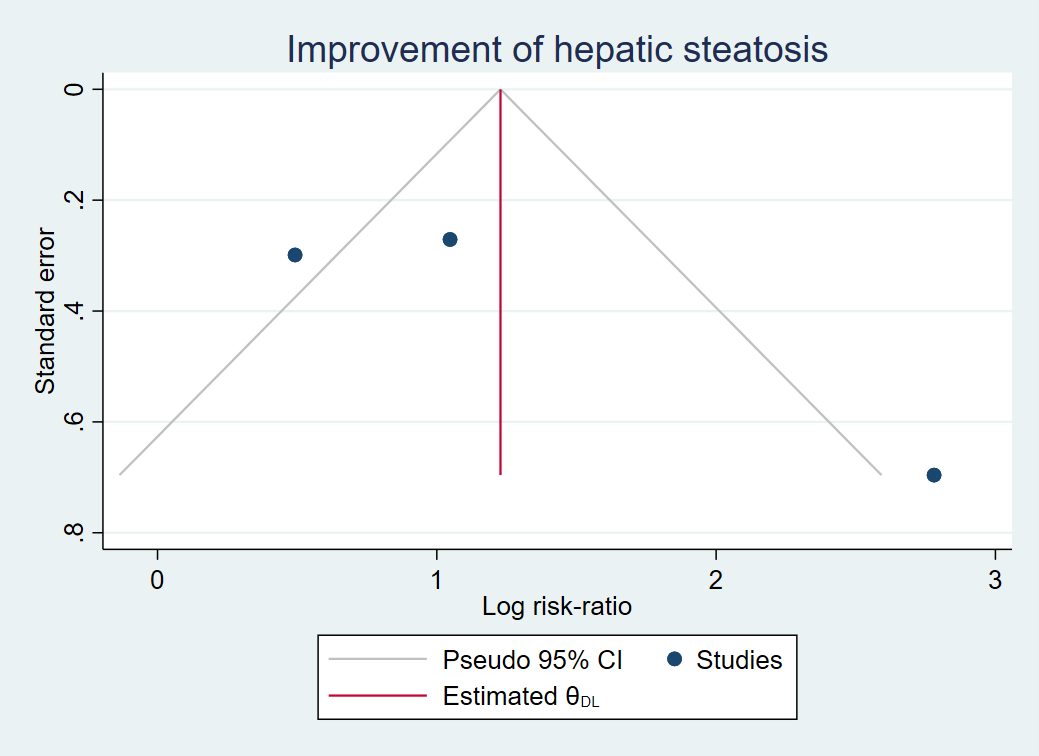


Appendix II. Contour-enhanced funnel plots of the improvement of hepatic steatosis


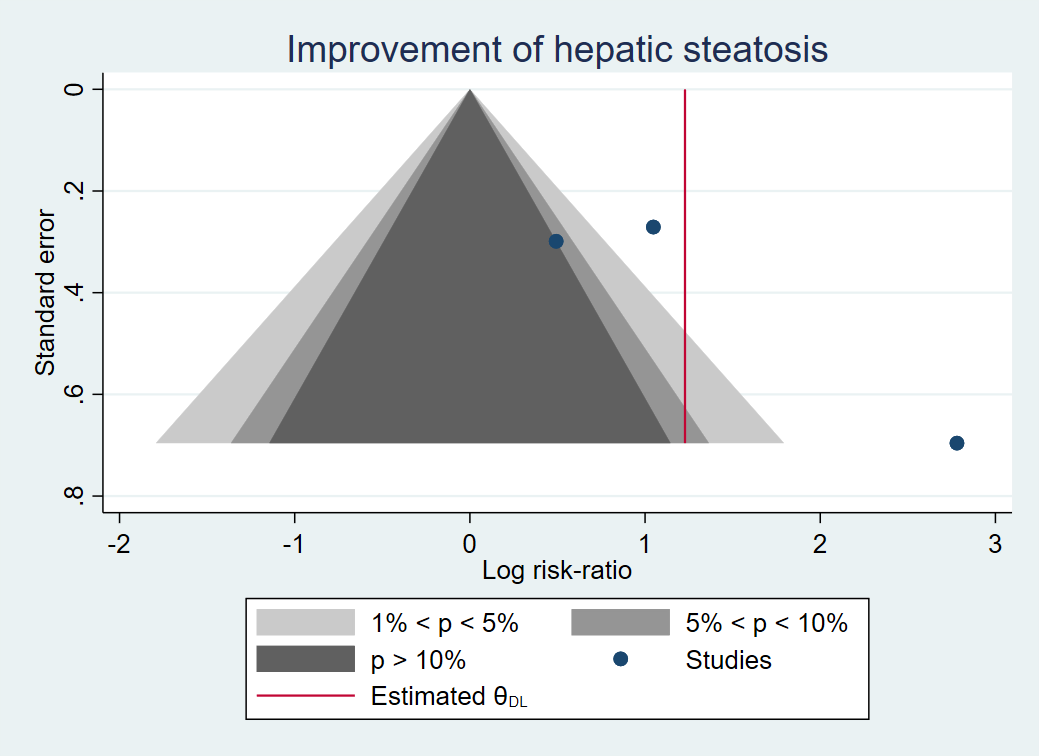


Appendix JJ. Funnel plots of fasting blood sugar


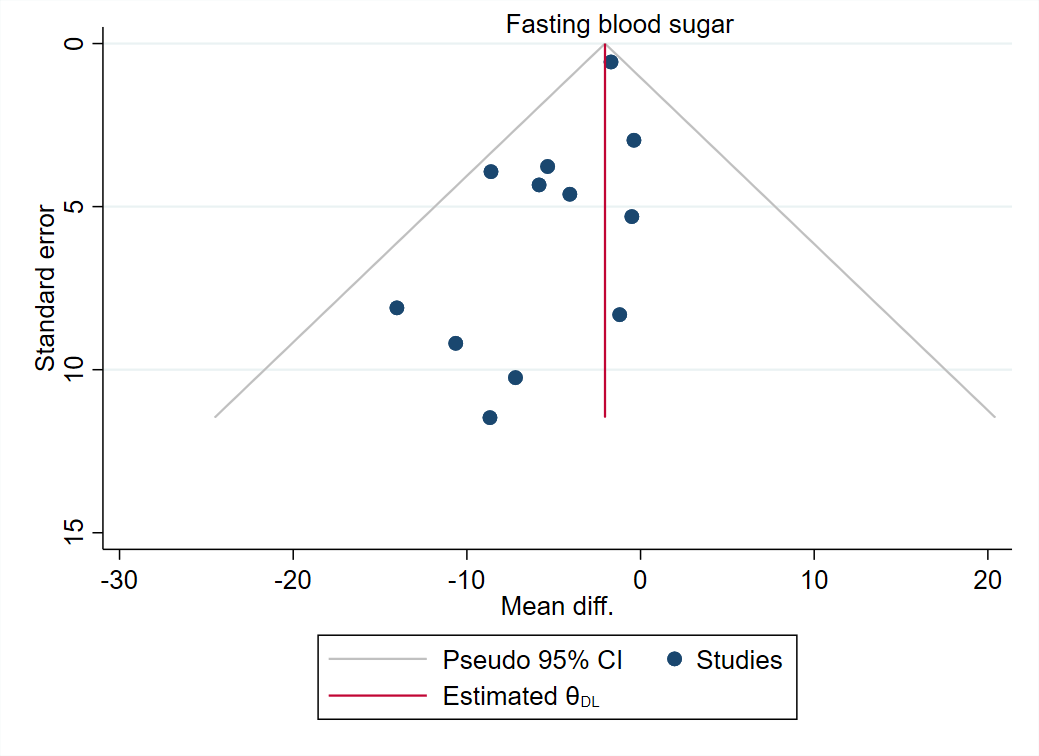


Appendix KK. Contour-enhanced funnel plots of fasting blood sugar


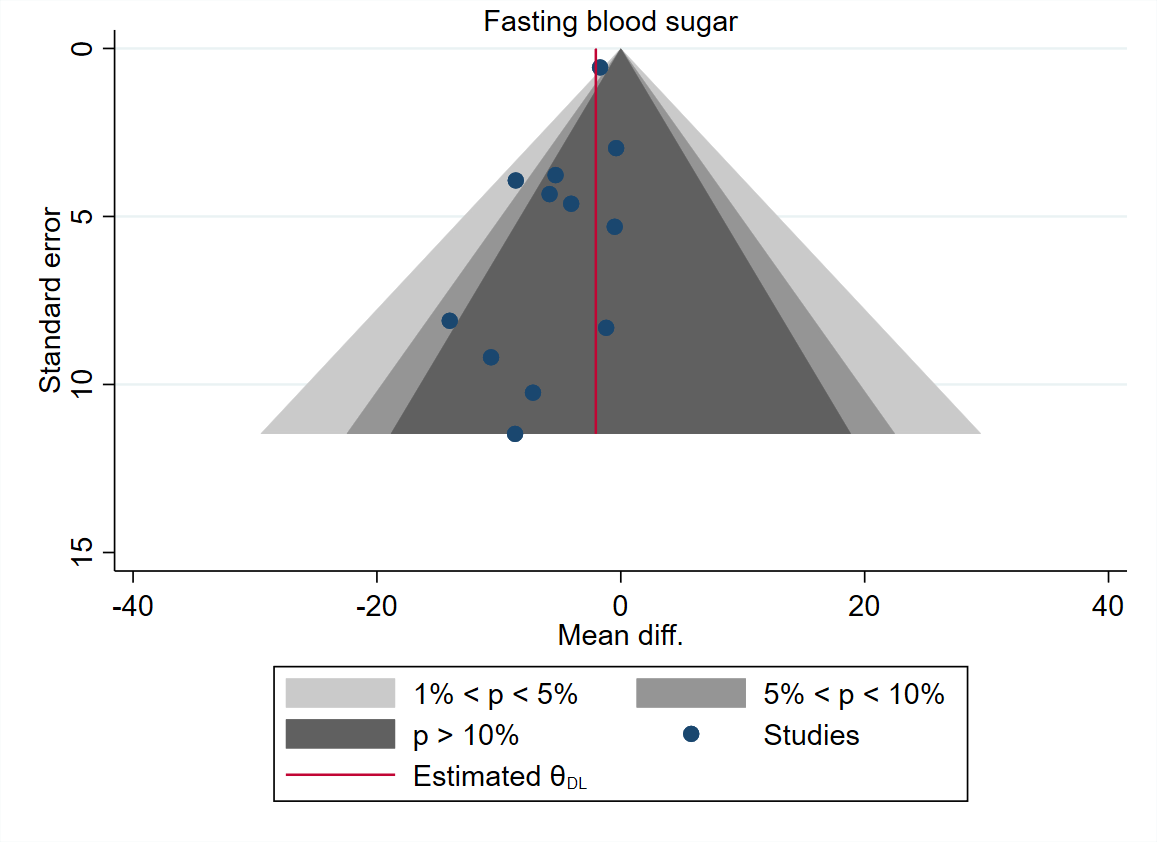


Appendix LL. Funnel plots of HbA1c


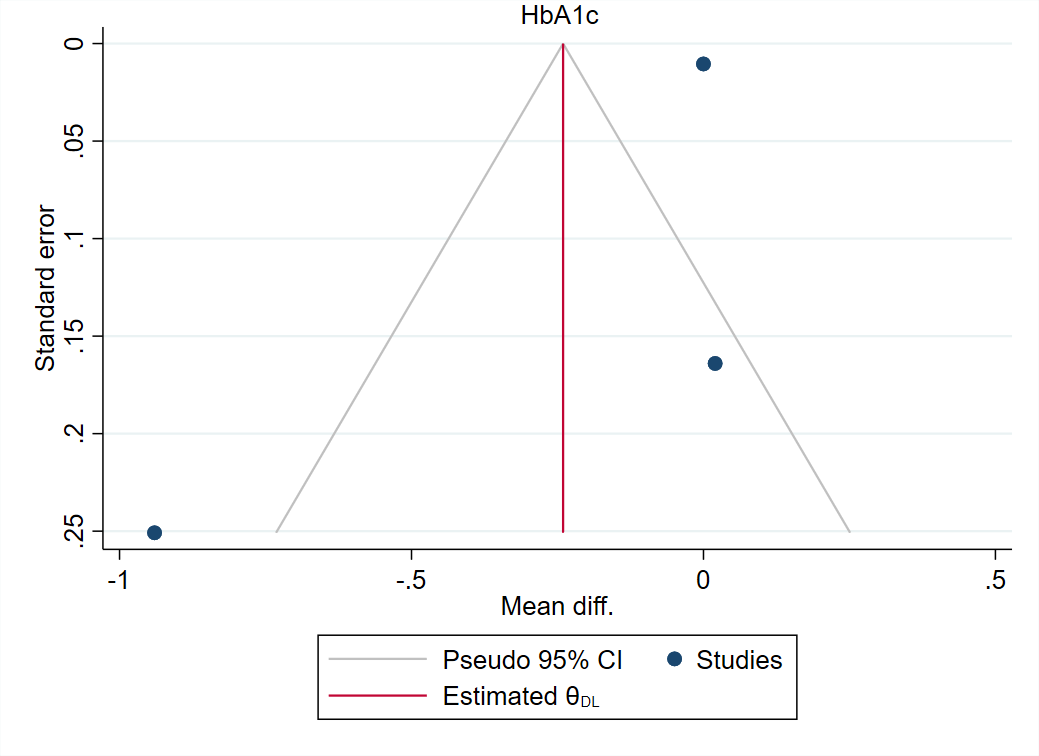


Appendix MM. Funnel plots of LDL-C

Appendix NN. Contour-enhanced funnel plots of LDL-C

Appendix OO. Funnel plots of HDL-C

Appendix PP. Contour-enhanced funnel plots of HDL-C

Appendix QQ. Funnel plots of SBP

Appendix RR. Contour-enhanced funnel plots of SBP

Appendix SS. Funnel plots of DBP

Appendix TT. Contour-enhanced funnel plots of DBP
